# SPP1/osteopontin: a driver of fibrosis and inflammation in degenerative ascending aortic aneurysm?

**DOI:** 10.1007/s00109-023-02370-z

**Published:** 2023-09-12

**Authors:** David Freiholtz, Otto Bergman, Sailendra Pradhananga, Karin Lång, Flore-Anne Poujade, Carl Granath, Christian Olsson, Anders Franco-Cereceda, Pelin Sahlén, Per Eriksson, Hanna M. Björck

**Affiliations:** 1 Section of Cardiothoracic Surgery, Department of Molecular Medicine and Surgery, Karolinska Institutet, Stockholm, Sweden; 2 Division of Cardiovascular Medicine, Center for Molecular Medicine, Department of Medicine, Karolinska Institutet, Stockholm, Karolinska University Hospital, Solna, Sweden; 3 KTH Royal Institute of Technology, School of Chemistry, Biotechnology and Health, Science for Life Laboratory, Tomtebodavägen 23A, 171 65, Stockholm, Sweden

**Keywords:** Aneurysm, Ascending aorta, Osteopontin, Fibrosis, Inflammation

## Abstract

**Abstract:**

Degenerative ascending aortic aneurysm (AscAA) is a silent and potentially fatal disease characterized by excessive vascular inflammation and fibrosis. We aimed to characterize the cellular and molecular signature for the fibrotic type of endothelial mesenchymal transition (EndMT) that has previously been described in degenerative AscAA. Patients undergoing elective open-heart surgery for AscAA and/or aortic valve repair were recruited. Gene expression in the intima-media of the ascending aorta was measured in 22 patients with non-dilated and 24 with dilated aortas, and candidate genes were identified. Protein expression was assessed using immunohistochemistry. Interacting distal gene enhancer regions were identified using targeted chromosome conformation capture (HiCap) in untreated and LPS-treated THP1 cells, and the associated transcription factors were analyzed. Differential expression analysis identified SPP1 (osteopontin) as a key gene in the signature of fibrotic EndMT in patients with degenerative AscAA. The aortic intima-media expression of *SPP1* correlated with the expression of inflammatory markers, the level of macrophage infiltration, and the aortic diameter. HiCap analysis, followed by transcription factor binding analysis, identified ETS1 as a potential regulator of SPP1 expression under inflammatory conditions. In conclusion, the present findings suggest that SPP1 may be involved in the development of the degenerative type of AscAA.

**Key messages:**

In the original manuscript titled “SPP1/osteopontin, a driver of fibrosis and inflammation in degenerative ascending aortic aneurysm?” by David Freiholtz, Otto Bergman, Saliendra Pradhananga, Karin Lång, Flore-Anne Poujade, Carl Granath, Christian Olsson, Anders Franco-Cereceda, Pelin Sahlén, Per Eriksson, and Hanna M Björck, we present novel findings on regulatory factors on osteopontin (SPP1) expression in immune cells involved in degenerative ascending aortic aneurysms (AscAA).

The central findings convey:

- SPP1 is a potential driver of the fibrotic endothelial-to-mesenchymal transition in AscAA.
- SPP1/osteopontin expression in AscAA is predominately by immune cells.
- ETS1 is a regulatory transcription factor of SPP1 expression in AscAA immune cells.

**Supplementary Information:**

The online version contains supplementary material available at 10.1007/s00109-023-02370-z.

## Introduction

Ascending aortic aneurysm (AscAA) is a progressive disease with an estimated incidence of 10 per 100,000 patient-years [1]. Although it is symptomatically silent, it is potentially fatal if dissection or rupture occurs [2]. AscAA has many different etiologies, including monogenic, aortopathy associated with a bicuspid aortic valve (BAV), and non-familiar degenerative AscAA developing in patients with normal tricuspid aortic valve (TAV) [3, 4]. Interestingly, the cellular trans-differentiation process referred to as endothelial mesenchymal transition (EndMT) has been implicated in the development of both BAV- and TAV-associated AscAA [5, 6], albeit that these conditions are characterized by completely different histomorphological aortic phenotypes. In BAV-associated AscAA, EndMT seems to manifest in the aortic intima-media, leaving the aortic wall structurally well-preserved. In TAV patients, on the other hand, EndMT-related changes are signified by medial degeneration [7], elastin fragmentation, excessive matrix component production, smooth muscle cell depletion, and, notably, inflammation [4, 8]. These are key features of a fibrotic type of EndMT, which interestingly also have been identified in abdominal aortic aneurysms (AAAs) [9, 10]. However, the specific molecular driver of the TAV-associated fibrotic EndMT is unknown. In the present study, we aimed to characterize the EndMT-related cellular and molecular signatures of degenerative AscAA and to identify regulatory factors that contribute to the signatures obtained.

## Material and methods

### Participants

Patients enrolled in the Advanced Study of Aortic Pathology (ASAP) [11] and Disease of the Aortic Valve, Ascending Aorta, and Coronary Artery (DAVAACA) [12, 13] cohorts were studied. All participants underwent elective open-heart surgery for aortic valve procedures, ascending aortic repair, or both at the Karolinska University Hospital, Stockholm, Sweden, from 2007 to 2023. Biopsies were collected from the anterior part of the proximal aorta, at the site of aortotomy, a few centimeters proximal to the aortic valve. Aortas with a maximal ascending aortic diameter of < 40 mm were classified as non-dilated (ND), and those with aortic diameters of > 45 mm were considered to be dilated (D). The exclusion criteria were syndromic aortic pathology and dissection. The study was approved by the Human Research Ethics Approval Committee of Stockholm (approval nos. 2006/784–31/1 and 2012/1633–31/4). Written informed consent was obtained from all the participants, and the study conformed to the principles of the Declaration of Helsinki. Expression analysis of the aortic intima-media was performed in samples from 111 participants, and immunohistochemistry was performed on samples from 36 participants (Supplementary Table S1).

### mRNA extraction and gene expression analysis

The intima-media layer of each aorta was separated from the adventitia and mRNA was extracted from the former using an RNeasy kit (Qiagen, Hilden, Germany), according to the manufacturer’s instructions. Global gene expression was then measured in 23 participants with TAV-ND, 21 with TAV-D, 31 with BAV-ND, and 44 with BAV-D using an Affymetrix GeneChip® Human Exon 1.0 ST array and the associated protocols, as previously described [7].

### Immunohistochemistry

Surgical biopsies of the aortas of 17–19 participants in the TAV-D and TAV-ND groups were embedded in paraffin and sectioned at a thickness of 5 µm using a microtome. Immunostaining for CD68 and osteopontin was performed on deparaffinized sections treated with Tris–EDTA solution (ab93684, Abcam, Cambridge, UK) using a mouse anti-CD68 antibody (M087601-2, Agilent DAKO, Santa Clara, CA, USA) and a rabbit anti-osteopontin antibody (ab283669, Abcam, Cambridge, United Kingdom) diluted 1:100. A goat anti-mouse Alexa Fluor™ 568 IgG (A-21090, Invitrogen, Waltham, MA, USA) and a goat anti-rabbit Alexa Fluor™ 647 IgG (A21244, Invitrogen, Waltham, MA, USA) were used as the secondary antibodies, which was diluted 1:600, and the sections were incubated for 2 h at room temperature. Nuclear counterstain with 4,6-diamidino-2-phenylindole (DAPI) (MBD0015, Sigma-Aldrich, St. Louis, MI, USA) was used at dilution 1:4000 for 2 min at room temperature prior to mounting in Fluoromount-G (00–4958-02, Invitrogen, Waltham, MA, USA). Tissue sections were then visualized with a laser scanning confocal microscope (Nikon), and digital images were collected. Channels were split, and the signal of fluorophore 568 nm was converted to green in Fiji (Version 2.9.0, ImageJ2).

### Cell culture and HiCap analysis

Human monocytic leukemia (THP-1) cells were cultured in conditioned RPMI1640 medium (Gibco 61,870–010) containing 10% fetal bovine serum (Gibco 10,106–169), 100 units/ml penicillin/100 μg/ml streptomycin (Gibco 15,140–122), and sodium pyruvate (Gibco 11,360–039). THP-1 cells were differentiated into macrophages using 100 ng/mL phorbol 12-myristate 13-acetate (PMA) (Sigma P8139) for 24 h, as previously described [14]. New medium was added to cells treated with PMA and they were incubated for a further 24 h to prepare conditioned medium. Then, new THP-1 cells were incubated for 24 h in the conditioned medium. To simulate an early inflammatory milieu, the THP-1-derived macrophages were subsequently incubated with new media in the absence of fetal bovine serum but containing 1 μg/μl LPS (Sigma L6529), for 2 h, and then washed with phosphate-buffered saline. The remaining THP-1-derived macrophages that had not been treated with LPS were used as controls [15, 16].

LPS-stimulated and unstimulated differentiated THP-1 cells were used for high-throughput chromosome conformation capture, followed by targeted chromosome conformation capture (HiCap), as previously described [15, 16]. Briefly, cells were fixed in a 1% formaldehyde solution, and then their nuclei were isolated. Chromatin was solubilized using sodium dodecyl sulfate and then enzymatically digested using 1 µg/µl FastDigest MboI (↓GATC; ThermoFisher Scientific). The Klenow fragment of DNA polymerase I and biotin-14-dATP were used to fill the protruding 5′-DNA strand ends left by the restriction enzyme. The product, with biotin-labeled blunt DNA ends, was then subjected to proximity ligation using T4 DNA ligase (New England Biolabs) for 4.5 h at 16 °C. Once the DNA that was spatially close by was ligated, proteinase K was used to aid the thermal removal of formaldehyde crosslinks. The 3′-5′ exonuclease activity of T4 DNA polymerase was used for 15 min at 12 °C to remove any unligated ends containing biotin.

Subsequently, sonication (Covaris Inc.) was used to fragment the chimeric DNA into 100–200 bp fragments, which were used with a KAPA HTP Library Preparation kit for Illumina platforms to prepare DNA sequencing libraries. To facilitate subsequent Illumina TruSeq LT adapter fusion, the ends of the fragments were repaired, and a poly-A tail was added to each. The manufacturer’s protocol was then modified by the addition of avidin–biotin selection of target DNA fragments using MyOne C1 streptavidin beads. The beads were washed and resuspended in water for the amplification of the prepared sequencing libraries. Thermal cycling was performed using an initial denaturation step of 45 s duration at 98 °C, followed by six cycles of denaturation (15 s at 98 °C), primer annealing (30 s at 60 °C), and strand extension (30 s at 72 °C), and a final single elongation step lasting 1 min at 72 °C. The obtained library was subjected to enrichment by custom target capture using the SureSelect XT Target Enrichment System for Illumina Paired-End Multiplexed Sequencing libraries (Agilent). The library was then hybridized under stringent conditions with a custom pre-designed RNA probe panel. Following this hybridization, the selected libraries were washed under stringent conditions to remove unhybridized DNA and then subjected to eight cycles of post-capture PCR, as described in the manufacturer’s protocol. The resulting enriched DNA libraries were purified and sequenced in-house using Illumina single index paired-end sequencing on a NextSeq 500 platform (Illumina Inc.).

### Transfection

Differentiated THP-1 cells were transfected using Lipofectamine RNAiMAX (Invitrogen 13,778–075, Concord, MA, USA), following manufacturer instructions. ETS1 Silencer Select Pre-Designed siRNA s4847 and s4849 (Thermo Fisher Scientific, #439,240 and negative control AM4611 (Thermo Fisher Scientifi) were used. In brief, siRNA and Lipofectamine RNAiMAX were diluted in Opti-MEM media (Thermo Fisher Scientific, #31,985–062), incubated for 20 min, and then administered to the cells for 24 h. Following transfection, cells were treated with LPS for 2 h, as described above, and then harvested. Each transfection reaction (*n* = 3) was performed in triplicate. RNA was extracted using a miRNeasy kit (217,004, Qiagen), and cDNA was synthesized from RNA using SuperScript III (Thermo Fisher Scientific, #18,080–051), in line with manufacturer’s protocol. TaqMan Universal Master mix (Thermo Fisher Scientific, #4,440,038) and SPP1 and ETS1 assays (Thermo Fisher Scientific, Hs00959010_m1 and Hs00428293_m1 for ETS1 and SPP1, respectively) were used for qPCR.

### Dissociation of aortic tissue

Aortic tissue dissociation was performed according to the protocol of Li et al. [17]. The aortic intima-media was separated from the adventitia by adventectomy, and 50-mg (per reaction) pieces of dilated and non-dilated aorta were placed in PBS − / − (Gibco 14,190–144). The tissue was dispersed in HBSS − / − (14,175,095, Thermo Fisher Scientific) containing 10% fetal bovine serum (10,270,106, Thermo Fisher Scientific); then placed in 10 ml of a solution containing 3 mg/ml collagenase type II (LS004176, Bionordika), 0.15 mg/ml collagenase type XI (C7657, Merck), 0.25 mg/ml soybean trypsin inhibitor (LS003571, BioNordika), 0.1875 mg/ml lyophilized elastase (LS002292, BioNordika), 0.24 mg/ml hyaluronidase type I (H3506, Merck), and 2.38 mg/ml HEPES (H3375, Merck) diluted in HBSS + / + (14,025,092, Thermo Fisher Scientific); and digested at 37 °C in a water bath for 1 h. The resulting cell suspensions were passed through a 40-μm cell strainer (CLS431750, Merck), centrifuged at 3000 × g for 2 min, and resuspended in CUT&RUN wash buffer (Cell Signaling #31,415).

### Confirmation of TF binding using CUT&RUN

The protein-DNA interaction of SPP1 with Distal 3 was analyzed using cleavage under targets and release using nuclease (CUT&RUN assay kit, Cell Signaling # 86,652) in differentiated THP-1 cells, treated or not with LPS, and dissociated aortic intima-media cells, following the Cell Signaling protocol (86,652) for unfixed live cells and tissue, with adjustments to render it suitable for use with dissected aorta. Approximately 200,000 cells were harvested per reaction from each of the two LPS-treated and two untreated samples. A rabbit monoclonal antibody (mAb) against trimethylated histone H3 (Lys4) (H3K4me3, 9751), included in the Cell Signaling kit, served as a positive control. An anti-ETS-1 antibody (14,069, Cell Signaling), diluted 1:50, was used for binding. DNA was purified using DNA purification buffers and spin columns (14,209, Cell Signaling), according to the manufacturer’s protocol. The amplified PCR products were visualized following agarose gel electrophoresis. The ETS-1 primer sequences were as follows: forward, AGGAGTGGTAAGCAAGGTAGG and reverse, GTCTGTTGAGCAACTTCCTCCTG, and an amplification temperature of 71 °C was used. The control primers (RPL30) provided by the manufacturer were also used for amplification at a temperature of 60 °C.

### Statistical analysis

Gene expression analysis was performed using the limma package in R [18]. HiCap data were processed and analyzed using in-house scripts in R using the GenomicRanges R package [19]. The CUT&RUN analyses were performed using the framework described by Ewels et al. [20]. Genomic data were visualized using the Integrative Genomics Viewer (IGV) [21]. All the analyses were conducted in the R environment (v4.1) and RStudio (v1.4) software. The relationships between continuous variables were evaluated using Pearson correlation in *R*. Figures were plotted using the ggplot2 package. The gene sets used in the functional enrichment analyses were retrieved from the “Molecular Signatures Database” (MSigDB, v7.5) using the msigdbr package [22].

## Results

### SPP1: a player in the fibrotic type of endothelial-to-mesenchymal transition?

**Fig. 1:**
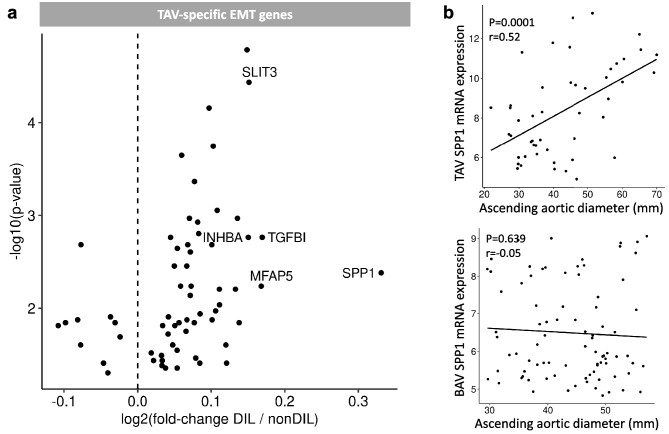
**a** EMT genes with differing expression in non-dilated and dilated ascending aortas from participants with TAV (i.e., TAV-specific dilatation genes). An alpha cut-off value of 0.5 was used. The log2 fold difference is shown on the x-axis, and the − log10 *p*-values for participants with dilated TAV (*n* = 24) and non-dilated TAV (*n* = 22) on the *y*-axis. **b** Correlation between the aortic intima-media mRNA expression of SPP1 and the ascending aortic diameters (mm), *P* = 0.0001, Pearson *r* = 0.52, (TAV *n* = 46, BAV *n* = 65). TAV, tricuspid aortic valve; BAV, bicuspid aortic valve

Degenerative ascending aortic aneurysms are characterized by fibrotic EndMT with an inflammatory component. To gain insight into the mechanism underpinning fibrotic EndMT, the expression of EMT-related genes, based on the Hallmark gene set in MedSig database, was analyzed in non-dilated and dilated ascending aortas from participants with BAV or TAV, respectively (Supplementary Fig. S1). Then, to identify the EndMT genes associated with TAV aortopathy, all the genes that were also upregulated in BAV dilated aortas were excluded, which yielded a final set of 63 TAV-specific EMT genes. Among these, *SPP1* showed the greatest fold difference in expression between non-dilated and dilated aortas (Fig. 1a) and was chosen for further investigation. Of note, *SPP1* expression correlated with the diameter (*p* = 0.0001) of the TAV ascending aortic intima-media, but not (*p* = 0.639) with that of BAV aortic intima-media (Fig. 1b). A complete list of the differentially expressed TAV-specific EMT genes is presented in Supplementary Table S2. A list of all the differentially expressed genes, with the magnitudes and directions of the differences, and their Hallmark classification are shown in Supplementary Table S3.

### SPP1/osteopontin expression correlates with indices of inflammation

**Fig. 2:**
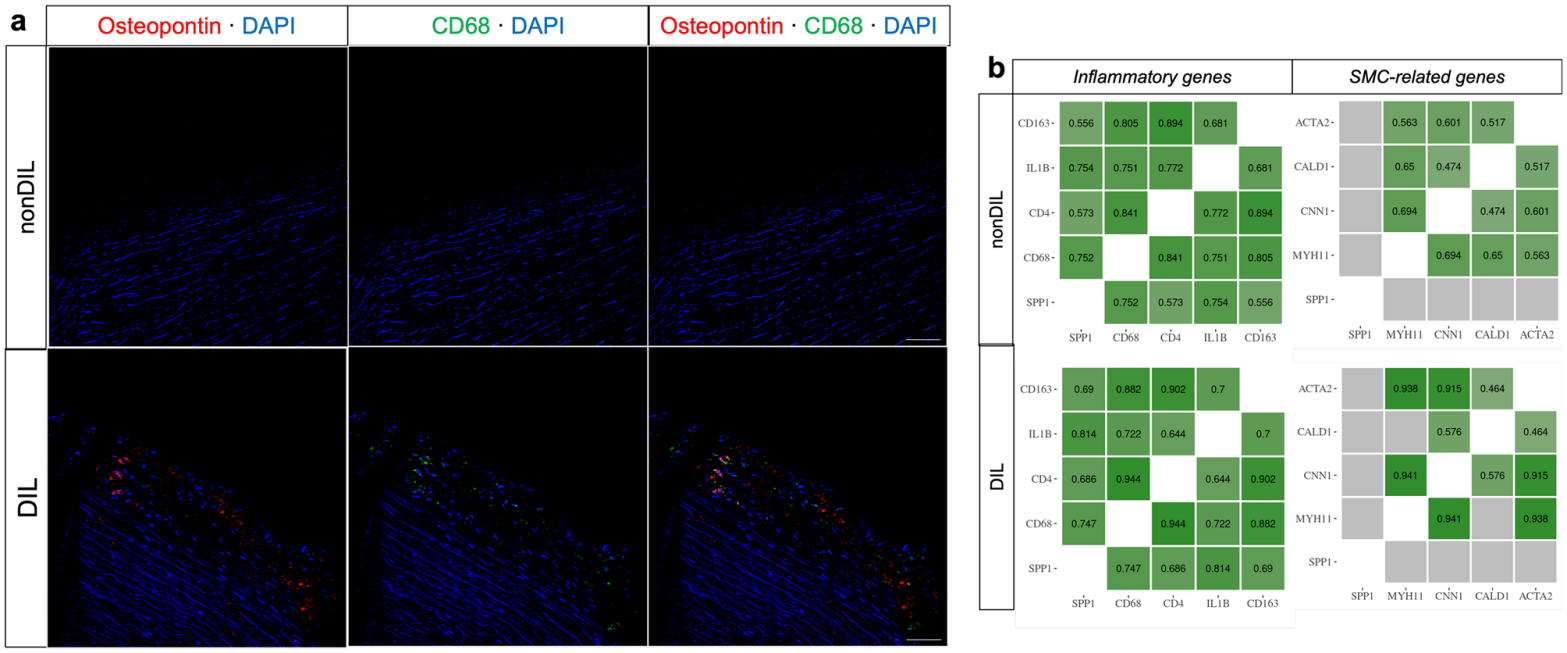
**a** Osteopontin and CD68 protein expression in participants with non-dilated (*n* = 19) or dilated (*n* = 17) ascending aortas with tricuspid aortic valves (magnification × 20, scale bar 20 μm). **b** Correlations of the gene expression of *SPP1* with inflammatory and immune cell markers (*IL1B*, *CD168*, *CD4*, and *CD163*) and smooth muscle cell markers (*ACTA2, CALD1, CNN1,* and *MYH11*) in non-dilated (*n* = 22) and dilated (*n* = 24) ascending aortas

**Fig. 3:**
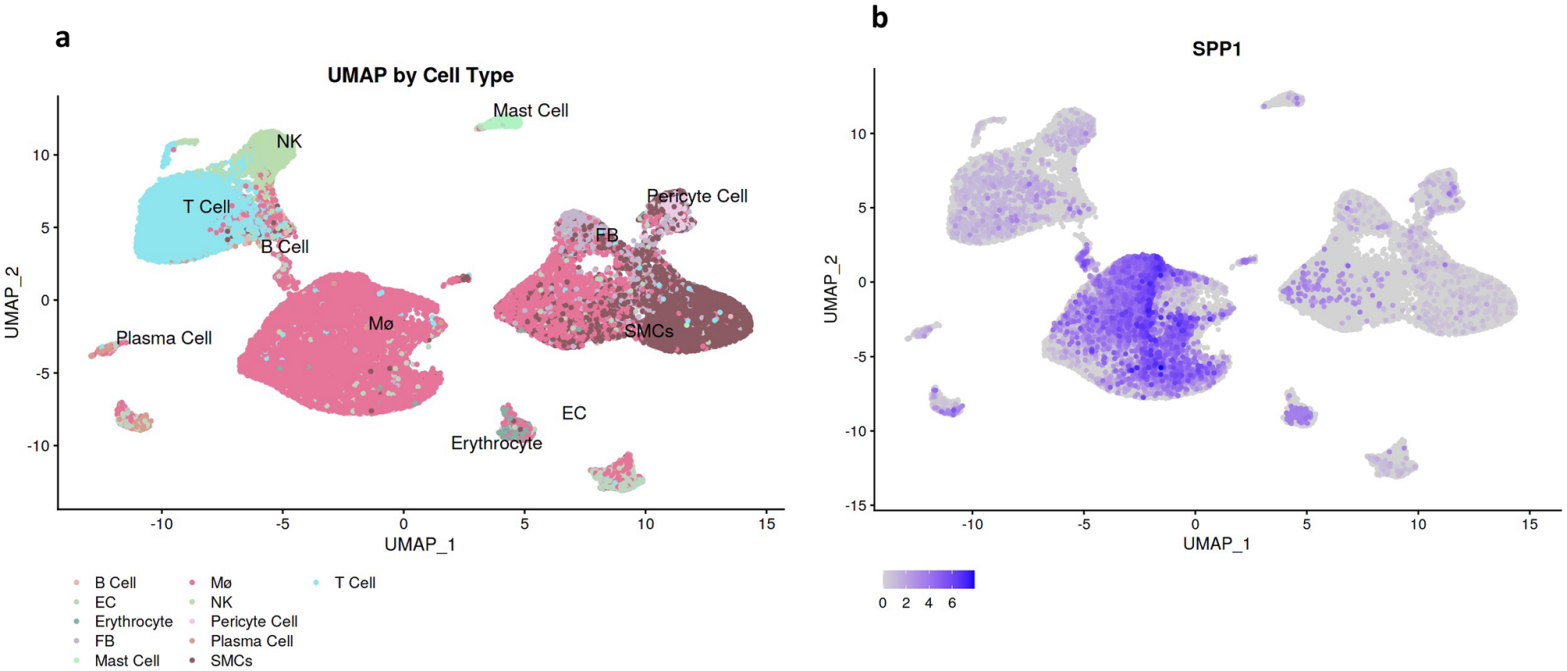
Single-cell genomic analysis of SPP1 expression in Plaqview 2.0 software [31], performed using publicly available data regarding thoracic aortic aneurysm tissue (Li et al. [21]). EC, endothelial cell; FB, fibroblast; NK, natural killer cell; SMCs, smooth muscle cells; Mø, macrophage

The localization and expression of the osteopontin protein in non-dilated and dilated TAV aortas were next characterized. As shown in Fig. 2a, non-dilated aortic tissue showed low osteopontin protein expression, but this was much higher in dilated aortas, which is in accordance with the *SPP1* mRNA expression data. Osteopontin expression was apparent in the subintima, media, and adventitia. Furthermore, analysis of a publicly available single-cell genomic dataset [17] using Plaqview 2.0 [23] software revealed that the *SPP1* mRNA expression was predominately in macrophages and T-cell clusters of degenerative AscAA tissue (Fig. 3). Similarly, to identify the potential source of osteopontin expression, we performed correlation analysis of *SPP1* mRNA expression with the expression of inflammatory and immune cell markers (*IL1B*, *CD68*, *CD4*, and *CD163*) and smooth muscle cell (SMC) markers (*ACTA2*, *MYH11*, *CNN1*, and *CALD1*). As shown in Fig. 2b, *SPP1* mRNA expression significantly correlated with *IL1B*, *CD168*, *CD4*, and *CD163* expression, but not with the expression of any of the SMC genes, which suggests that immune cells are the primary source of osteopontin SPP1 expression in TAV ascending aortas. These data were corroborated by the partially overlapping expression pattern of osteopontin and CD68 protein in the ascending aortic intima of dilated tissue samples, while nearly absent in non-dilated samples (Fig. 2a).

### ETS1 as a possible regulator of SPP1 expression

**Fig. 4:**
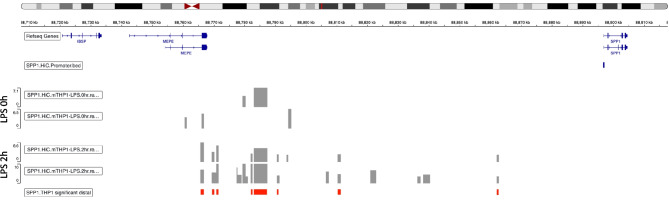
Results of the HiCap analysis of SPP1 promoter interactions in THP1 cells stimulated or not with LPS. The results are mapped to their chromosomal positions. Gray bars indicate distal elements interacting with the SPP1 promoter. The samples were analyzed in duplicate, and the distal elements identified in both samples with a significance of *p* < 0.001 were considered to show significant interactions (red bars)

To further understand the regulation of SPP1 expression and identify putative transcription factors, HiCap analysis of the SPP1 promoter was performed in differentiated THP1 cells. To trigger an inflammatory response, the cells were activated by LPS treatment for 2 h, and the distal elements interacting with the SPP1 promoter (gray bars) were identified under unstimulated and stimulated conditions (Fig. 4). High-resolution chromatin interaction maps were analyzed to identify active enhancers that regulate SPP1 expression under proinflammatory conditions [16]. Of note, the samples were analyzed in duplicate and only the distal elements identified in both samples were considered to represent significant interactions (red bars). In total, eight distal elements were identified.

**Fig. 5:**
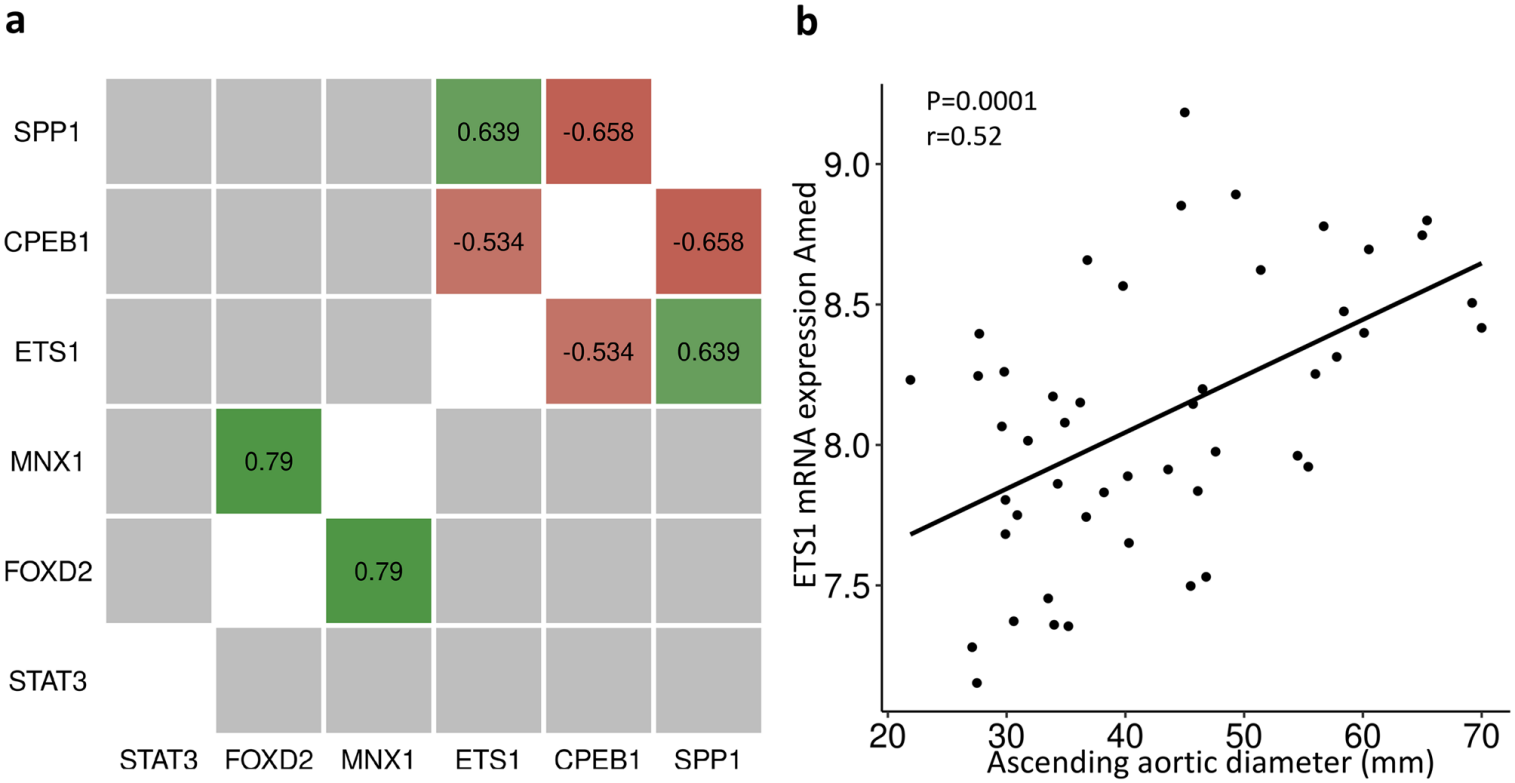
**a** Correlations of SPP1 expression with that of the top five putative transcription factors that were differentially expressed in non-dilated and dilated ascending aortas. Red boxes indicate negative correlations; green boxes indicate positive correlations, and gray boxes indicate non-significant relationships. Pearson *r*-values are shown; *n* = 22. **b** Correlation between the aortic intima-media mRNA expression of ETS1 and the ascending aortic dimensions (mm). *p* = 0.0001, Pearson *r* = 0.52, *n* = 46

Next, TRANSFAC® was used to identify putative transcription factor-binding sites (TFBS) within each distal element, and this was followed by differential mRNA expression analysis of putative transcription factors (TFs) in the samples of ascending aorta. A complete list of putative transcription factors is shown in Supplementary Fig. S2, and the significantly differentially expressed (DE) TFs are shown in Supplementary Table 4 (alpha level 0.5). Then, to prioritize the TFs that might be important for the regulation of SPP1 expression, the relationships between the expression of DE TFs and *SPP1* mRNA expression were assessed using correlation analysis (Fig. 5a). We found that the expression of only one of the predicted TFs correlated positively with that of *SPP1*: *ETS1* (Pearson *r* = 0.64, *p* ≤ 0.001). Consistent with this, aortic intima-media *ETS1* mRNA expression positively correlated with the dimensions of the ascending aorta (*p* = 0.0001, Pearson *r* = 0.52) (Fig. 5b). The expression of one predicted TF, CPEB1, inversely correlated with that of *SPP1* (Pearson *r* =  − 0.66, *p* < 0.001 (Fig. 5a).

**Fig. 6:**
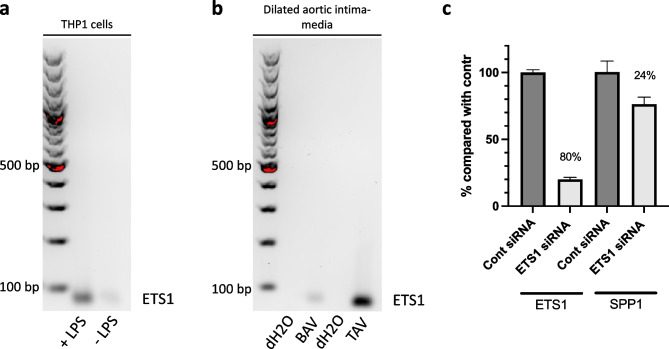
TF binding analysis using the CUT&RUN-method for the interaction between SPP1 and ETS1 in THP1 cells, treated with LPS (2 h) or not (0 h) (**a**) and in dilated ascending aortas (**b**). ETS1 binding is demonstrated using a 2% agarose gel. **c** *ETS1* and *SPP1* mRNA expression in differentiated THP1 cells transfected with ETS1 and control siRNA, respectively, prior to 2 h LPS stimulation

To validate our HiCap results and the finding that ETS1 is a potential regulator of SPP1 expression, the binding of ETS1 to distal region 3, which interacts with the SPP1 promoter under LPS-treated conditions, was investigated using CUT&RUN analysis. This showed that LPS stimulation did indeed markedly increase ETS1 binding to the putative enhancer that interacts with the SPP1 promoter (Fig. 6a). The binding of ETS1 to distal region 3 was further confirmed in dilated aortic intima-media samples from participants with TAV (Fig. 6b). Moreover, transfection experiments further supported ETS1 as a transcriptional regulator of SPP1, showing a reduced *SPP1* mRNA expression in differentiated THP1 cells treated with ETS1 siRNA prior to LPS stimulation (Fig. 6c). *SPP1* mRNA expression was reduced by an average of 24% (0.76 ± 0.09) in cells treated with ETS1 siRNA compared with cells treated with control siRNA.

### ETS1 expression correlates with the expression of proinflammatory genes in TAV aortic intima-media

To gain a broader understanding of the potential role of ETS1 in the development of aneurysm, we screened aortic intima-media samples from participants with TAV for TAV-specific co-expression with *ETS1*. As shown in Table 1, the genes whose expression positively correlated with that of *ETS1* frequently encoded proteins involved in immune system pathways (innate, FDR *q* = 2.7E − 19; adaptive, FDR *q* = 5.5E − 15) and cytokine signaling in the immune system (FDR *q* = 1.8E − 11). Of note, the immune- and inflammation-associated profile associated with TAV dilation was further strengthened when other DE genes were analyzed on the basis of fold change, during which genes such as *CLEC5A*, *PTPRC*, *OLR1*, *KRT18*, *CCR2*, *TLR7*, and *MSR1* were identified.

**Table 1:** Enrichment analysis of genes correlating with aortic intima-media ETS1 expression in patients with tricuspid aortic valves

Reactome pathway	No. of genes	*p*-value
Innate immune system	43	2.72E − 19
Adaptive immune system	33	5.36E − 15
Hemostasis	27	4.33E − 12
Cytokine signaling in the immune system	27	1.84E − 11
Neutrophil degranulation	22	5.35E − 11
Infectious disease	27	9.05E − 8
Cell surface interactions at the vascular wall	12	5.15E − 7
Signaling by interleukins	17	8.82E − 7
Platelet activation, signaling, and aggregation	13	1.17E − 6

## Discussion

Degenerative aneurysms of the ascending aorta are characterized by progressive deterioration, involving smooth muscle cell death, immune cell infiltration, and remodeling of the extracellular matrix[24]. Along with fibrosis, this represents a pathological signature of the progressive type II EndMT. In the present study, we performed a molecular characterization of TAV-associated AscAA and identified SPP1 as a potentially important component of the EndMT signature associated with the development of AscAA. Specifically, the aortic intima-media expression of *SPP1* was markedly upregulated, and upon further investigation, its expression was found to be associated with an inflammatory expression profile and the presence of macrophages. Using HiCap [25], we identified ETS1 as a potential transcriptional regulator of *SPP1* expression and confirmed its binding to the SPP1 promoter in differentiated THP-1 cells under inflammatory conditions, as well as in samples of dilated ascending aorta from patients with TAV. Taken together, these data suggest a role for ETS1, with subsequent upregulation of SPP1 in macrophages infiltrating the aneurysmal site, in the pathology of degenerative AscAA.

The present results confirm that inflammation is a key component of non-familiar degenerative ascending aortopathy. In both thoracic and abdominal aortic aneurysms (AAA), immune cell infiltration and matrix degeneration are key processes associated with dilation, as is a switch in the phenotype of vascular smooth muscle cells toward a fibroblast-like phenotype [4, 10, 26]. In AAA, it has been proposed that matrix degradation per se promotes inflammation and the recruitment of leukocytes to aneurysms, thereby increasing protease activity and cellular trans-differentiation [27, 28]. In AscAA, the factors initiating the inflammatory process are unknown.

Fibrosis and EndMT have previously been noted in both TAA [4, 29] and AAA [8–10]. Here, we show that the expressions of several collagen genes (e.g., *COL1A1*, *COL12A1*, *COL4A1*, *COL4A2*, and *COL3A1*) were up-regulated in dilated ascending aortas, further supporting this signature.

SPP1, more frequently known as osteopontin, has previously been implicated in degenerative aneurysmal disease, in particular AAA [30], and shown to play a role in various processes in blood vessels [31, 32]. For example, the SPP1 that is secreted during inflammation binds to transmembrane ligands and regulates tissue remodeling pathways [33]. Moreover, SPP1 is involved in cellular migration, proliferation, apoptosis, and macrophage chemotaxis [34]. Previous studies have also implicated SPP1 in both abdominal and thoracic aneurysmal disease by upregulating matrix proteinases via NF-κB, thereby accelerating tissue degeneration [35]. In addition, in SPP1-null mice, there is evidence of less leukocyte infiltration and lower matrix proteinase activity at the sites of aneurysms [34, 36]. The source of SPP1, however, seems to differ in AscAA and AAA. In AAA, vascular smooth muscle cells are the main source of *SPP1* expression [30], but in the dilated ascending aorta, we found that *SPP1* expression correlates with the expression of inflammatory markers and not with markers of smooth muscle cells. In addition, an immunohistochemical evaluation suggested that CD68-positive cells are a major source of osteopontin in dilated aortic tissue, through which it may drive tissue remodeling. This is consistent with the growing evidence that AscAA is characterized by inflammation [37, 38], with large numbers of T-lymphocytes and macrophages being present in the media and adventitia of thoracic aortic aneurysms [39, 40]. The upregulation of *SPP1* appears to occur alongside inflammation in EndMT, potentially accelerating disease progression. Furthermore, in a recent study, we showed that aspirin treatment is associated with a lower prevalence of AscAA in a surgical cohort, which may be explained by lower intima-media cyclic oxygenase 2 expression [13] and possibly immunomodulation.

Using HiCap analysis, ETS1 was identified as a potential regulator of SPP1 expression in response to inflammation, and their binding was demonstrated in cell culture and ex vivo. ETS1 belongs to the ETS family of TFs, with well-documented roles in the regulation of inflammation [41]. Interestingly, ETS1 has previously been implicated in human intracranial aneurysm (IA) formation, suggestively by mediating monocyte chemoattractant protein 1 expression in vascular smooth muscle cells to promote inflammation [42]. Also, inhibition of ETS1 decreased IA size and IA wall thickness in rats [43], further supporting the role of ETS1 in IA development. Although the specific molecular players driving aneurysm in intracranial arteries and the aorta may differ, it seems likely that ETS1 has a role also in the formation of aneurysm in other arterial segments. Indeed, it has previously been shown that ETS1-null mice present with decreased perivascular fibrosis and a smaller number of infiltrating leukocytes to the aortic wall following angiotensin II infusion [36]. Moreover, vascular injury has been associated with a high ETS1 expression [41], and the simultaneous inhibition of ETS1 and NF-κB in a rabbit model of AAA preserves elastin integrity and reduces aneurysm size [44]. Collectively, this suggests ETS1 as an important driver of multiple degenerative processes in blood vessels by promoting inflammation and arterial remodeling.

ETS1 also interacts with several molecules of the TGF-B pathway, including SMADs. For example, ETS1 enhances the transcriptional activity of SMAD3 and interacts with SMAD2[45], consequently leading to an increased TGF-β signaling. TGF-β is known for its involvement in syndromic forms of AscAA [46], and dysregulation of this pathway promotes fibrosis, inflammation, and EndMT[47]. Interestingly, the miR-200 family not only plays an important role in the regulation of the TGF-β pathway [48], but also directly targets ETS1 to repress the ETS-1-induced EMT [49]. Moreover, it was previously shown that ETS1 could reverse effects attributed to miR-590 to activate TGF-β in HUVECs treated with ox-LDL [50].

## Conclusion

In the present study, we have identified a cellular signature and its regulators in aortas from patients with AscAA. Specifically, SPP1, regulated by ETS1, may play a role in the progression of AscAA with a degenerative phenotype. These findings complement the growing body of literature regarding the role of inflammation and specific immune cells in degenerative AscAA and suggest a role for immunomodulation in the treatment or prevention of AscAA and its progression.

### Supplementary Information

Below is the link to the electronic supplementary material.

Supplementary file1 (DOCX 1759 KB)

## Data Availability

The datasets used and/or analyzed in the current study are available from the corresponding author upon reasonable request.
