# Supplementary material for: SPP1/osteopontin: a driver of fibrosis and inflammation in degenerative ascending aortic aneurysm?
Source: J Mol Med (Berl). 2023 Sep 12;101(10):1323–33. doi: 10.1007/s00109-023-02370-z (PMC10560177; doi:10.1007/s00109-023-02370-z)

**Supplementary materials**

**SPP1/osteopontin, a driver of fibrosis and inflammation in degenerative ascending aortic aneurysm?**

Short title: SPP1 and degenerative ascending aortic aneurysm

David Freiholtz, Otto Bergman, Sailendra Pradhananga, Karin Lång, Flore-Anne Poujade, Carl Granath, Christian Olsson, Anders Franco-Cereceda, Pelin Sahlén, Per Eriksson, Hanna M Björck

**Page Content**

2 Supplementary Table S1, Patient characteristics

3 Supplementary Table S2, Differentially expressed TAV-specific genes within the EMT hallmark, sorted by fold change

4 Supplementary Table S3, TAV-specific differentially expressed genes, sorted by fold-change and hallmark

22 Supplementary Table S4, Differentially expressed putative transcription factors (aortic intima-media) within HiCap distals, sorted by fold-change.

24 Supplementary Figure S1, Enrichment analysis of differentially expressed genes

25 Supplementary Figure S2, Putative transcription factors (TF) within each HiCap-identified distal

**Supplementary Table S1. Patient characteristics.** TAV-patients, immunohistochemical and gene expression analyses, respectively.


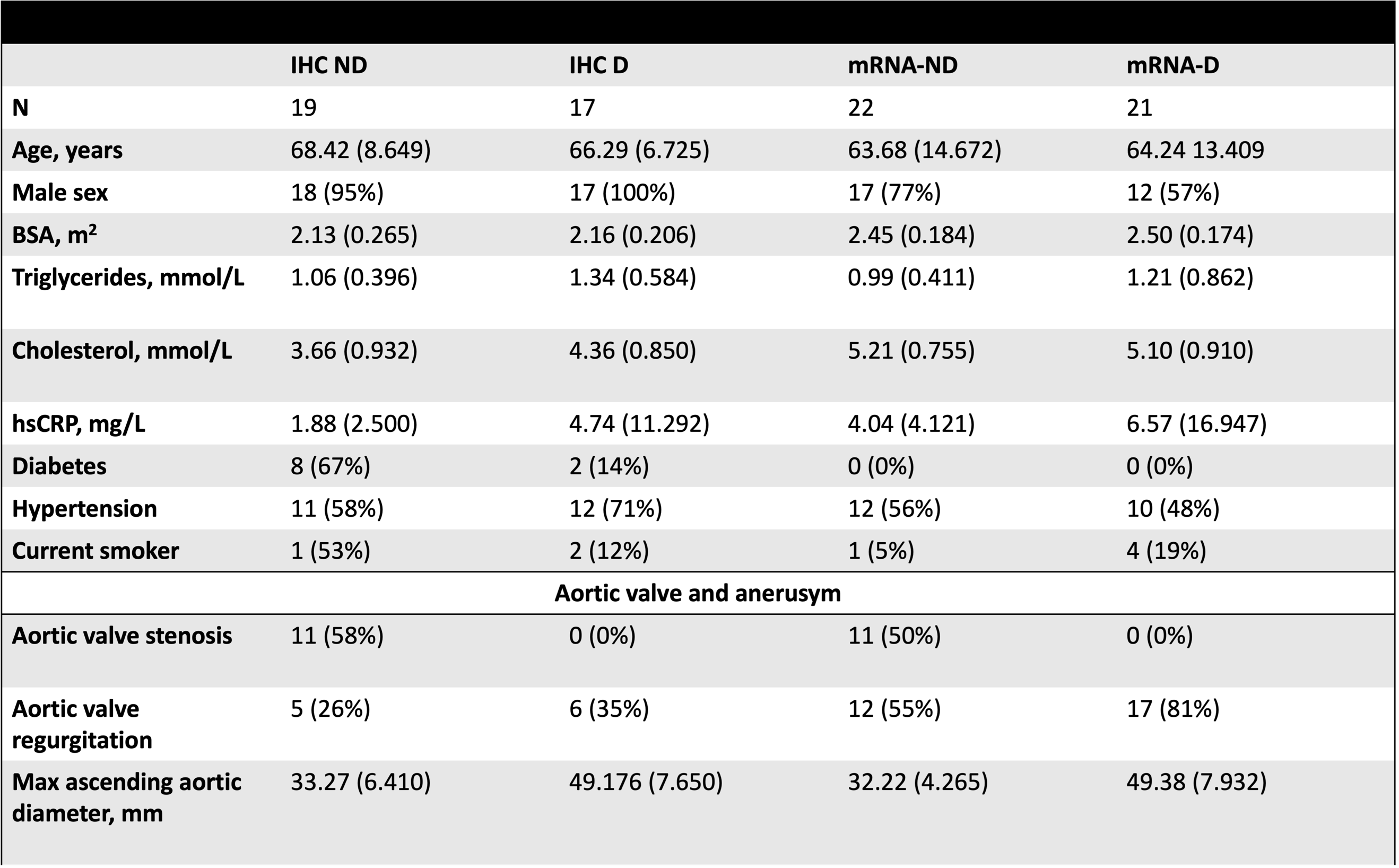


BSA, body surface area; TAV, Tricuspid aortic valve; hsCRP, high-sensitive C-reactive protein; IHC, Immunohistochemistry. Continuous variables are presented as mean (SD) and ordinal variables are presented as n (%). Non-dilated (ND) aorta <40mm; Dilated (D) aorta >45mm.

**Supplementary Table S2.** Differentially expressed TAV-specific genes within the EMT hallmark, sorted by fold change.

| **Gene (Hallmark EMT)** | **p (DIL/nonDIL)** | **Fc (DIL/nonDIL)** |
| --- | --- | --- |
| SPP1 | 0,00414 | 1,25784 |
| TGFBI | 0,0017202 | 1,12443869 |
| MFAP5 | 0,00574908 | 1,12335937 |
| SLIT3 | 3,69E-05 | 1,11057494 |
| INHBA | 0,0017202 | 1,10988479 |
| COL1A1 | 1,65E-05 | 1,10856655 |
| POSTN | 0,01430322 | 1,10029091 |
| COL12A1 | 0,00107314 | 1,0985889 |
| MXRA5 | 0,00622855 | 1,09651934 |
| NID2 | 0,03872779 | 1,08740263 |
| VEGFA | 0,02479368 | 1,08677222 |
| LAMA3 | 0,00919046 | 1,08025583 |
| FAP | 0,00622855 | 1,08011357 |
| CDH6 | 0,00088285 | 1,07784695 |
| THBS1 | 0,0106816 | 1,07623555 |
| COL4A1 | 0,00017984 | 1,07390517 |
| IGFBP3 | 0,01331078 | 1,07269547 |
| ECM1 | 0,00206457 | 1,0724076 |
| FBN1 | 6,96E-05 | 1,06969201 |
| LOXL2 | 0,01150289 | 1,06062648 |
| ITGA2 | 0,03872779 | 1,06037215 |
| PLAUR | 0,00156811 | 1,05920928 |
| MMP14 | 0,0011815 | 1,05828556 |
| PCOLCE | 0,03420622 | 1,05637329 |
| COL4A2 | 0,00043248 | 1,05508616 |
| THBS2 | 0,01430322 | 1,05485362 |
| COL3A1 | 0,00574908 | 1,05180907 |
| COL5A1 | 0,00246929 | 1,0510298 |
| ECM2 | 0,00729386 | 1,05092362 |
| ITGA5 | 0,00107314 | 1,05006536 |
| CDH11 | 0,00206457 | 1,04843016 |
| FAS | 0,01331078 | 1,04760249 |
| TGFB1 | 0,00349658 | 1,04749528 |
| COL6A3 | 0,01767178 | 1,04680198 |
| SERPINH1 | 0,00022542 | 1,04244442 |
| PLOD1 | 0,00574908 | 1,04144263 |
| ADAM12 | 0,01430322 | 1,03988828 |
| LRP1 | 0,00225885 | 1,0382088 |
| SAT1 | 0,04373476 | 1,03802535 |
| TNFAIP3 | 0,02825584 | 1,03797485 |
| CRLF1 | 0,01535873 | 1,03584508 |
| COL1A2 | 0,00349658 | 1,03535705 |
| COL5A2 | 0,02479368 | 1,0337041 |
| TPM1 | 0,0017202 | 1,03159226 |
| VCAN | 0,01237833 | 1,02958797 |
| CALD1 | 0,01893601 | 1,0292881 |
| ELN | 0,04373476 | 1,02673626 |
| PLOD3 | 0,01535873 | 1,02403996 |
| BMP1 | 0,03640861 | 1,02364137 |
| QSOX1 | 0,04116831 | 1,0233951 |
| FN1 | 0,0321162 | 1,02293514 |
| BGN | 0,03640861 | 1,01526119 |
| PVR | 0,03013416 | 1,01311388 |
| MCM7 | 0,02027666 | 0,98348978 |
| FOXC2 | 0,01430322 | 0,97890048 |
| MSX1 | 0,01237833 | 0,9747123 |
| AREG | 0,04926421 | 0,97228309 |
| GADD45A | 0,03872779 | 0,96830017 |
| PTHLH | 0,00206457 | 0,94803638 |
| PCOLCE2 | 0,02479368 | 0,94774204 |
| FBN2 | 0,01331078 | 0,9451106 |
| COMP | 0,01430322 | 0,93438877 |
| LAMA2 | 0,01535873 | 0,92801915 |

Non-dilated (nonDIL) aorta <40mm; Dilated (DIL) aorta >45mm.

**Supplementary Table S3.** TAV-specific differentially expressed genes, sorted by fold-change and hallmark.

| **Gene** | **p (DIL/nonDIL)** | **Fc (DIL/nonDIL)** | **Hallmark** |
| --- | --- | --- | --- |
| CLEC5A | 0,00143 | 1,31560 | INFLAMMATORY_RESPONSE |
| SPP1 | 0,00414 | 1,25784 | ANGIOGENESIS |
| SPP1 | 0,00414 | 1,25784 | IL2_STAT5_SIGNALING |
| SPP1 | 0,00414 | 1,25784 | KRAS_SIGNALING_UP |
| SPP1 | 0,00414 | 1,25784 | EPITHELIAL_MESENCHYMAL_TRANSITION |
| PTPRC | 0,00172 | 1,21040 | ALLOGRAFT_REJECTION |
| PTPRC | 0,00172 | 1,21040 | ALLOGRAFT_REJECTION |
| RGS1 | 0,00919 | 1,20920 | INFLAMMATORY_RESPONSE |
| KRT18 | 0,00391 | 1,19570 | ESTROGEN_RESPONSE_EARLY |
| C3 | 0,00575 | 1,18999 | COMPLEMENT |
| OLR1 | 0,00035 | 1,18766 | ANGIOGENESIS |
| OLR1 | 0,00035 | 1,18766 | COMPLEMENT |
| OLR1 | 0,00035 | 1,18766 | INFLAMMATORY_RESPONSE |
| CCR2 | 0,03641 | 1,18615 | ALLOGRAFT_REJECTION |
| CASP1 | 0,04117 | 1,18257 | COMPLEMENT |
| CASP1 | 0,04117 | 1,18257 | INTERFERON_GAMMA_RESPONSE |
| TLR7 | 0,00020 | 1,18113 | IL2_STAT5_SIGNALING |
| MSR1 | 0,00247 | 1,17354 | INFLAMMATORY_RESPONSE |
| CXCR4 | 0,00414 | 1,17054 | KRAS_SIGNALING_UP |
| GZMK | 0,02479 | 1,16922 | COMPLEMENT |
| GPR65 | 0,00009 | 1,16254 | ALLOGRAFT_REJECTION |
| GPR65 | 0,00009 | 1,16254 | IL2_STAT5_SIGNALING |
| BCAT1 | 0,00294 | 1,15987 | ALLOGRAFT_REJECTION |
| BCAT1 | 0,00294 | 1,15987 | XENOBIOTIC_METABOLISM |
| C3AR1 | 0,00226 | 1,15238 | INFLAMMATORY_RESPONSE |
| C3AR1 | 0,00226 | 1,15238 | KRAS_SIGNALING_UP |
| LCP1 | 0,00320937 | 1,14778048 | KRAS_SIGNALING_UP |
| HLA-DMB | 0,00320937 | 1,14773398 | ALLOGRAFT_REJECTION |
| HLA-DMB | 0,00320937 | 1,14773398 | ALLOGRAFT_REJECTION |
| HLA-DMB | 0,00320937 | 1,14773398 | ALLOGRAFT_REJECTION |
| HLA-DMB | 0,00320937 | 1,14773398 | ALLOGRAFT_REJECTION |
| HLA-DMB | 0,00320937 | 1,14773398 | ALLOGRAFT_REJECTION |
| HLA-DMB | 0,00320937 | 1,14773398 | ALLOGRAFT_REJECTION |
| HLA-DMB | 0,00320937 | 1,14773398 | ALLOGRAFT_REJECTION |
| HLA-DMB | 0,00320937 | 1,14773398 | ALLOGRAFT_REJECTION |
| F13A1 | 0,02479368 | 1,14359949 | KRAS_SIGNALING_UP |
| CXCL10 | 0,02169734 | 1,14347889 | IL2_STAT5_SIGNALING |
| CXCL10 | 0,02169734 | 1,14347889 | INFLAMMATORY_RESPONSE |
| CXCL10 | 0,02169734 | 1,14347889 | INTERFERON_GAMMA_RESPONSE |
| CXCL10 | 0,02169734 | 1,14347889 | KRAS_SIGNALING_UP |
| CTSS | 0,00851538 | 1,14296532 | ALLOGRAFT_REJECTION |
| CTSS | 0,00851538 | 1,14296532 | COMPLEMENT |
| CTSS | 0,00851538 | 1,14296532 | KRAS_SIGNALING_UP |
| KLRK1 | 0,0017202 | 1,14235717 | INTERFERON_GAMMA_RESPONSE |
| PLEK | 0,00320937 | 1,14134832 | COMPLEMENT |
| LAPTM5 | 0,00349658 | 1,14057199 | KRAS_SIGNALING_UP |
| ST8SIA4 | 0,00107314 | 1,13924862 | ALLOGRAFT_REJECTION |
| ST8SIA4 | 0,00107314 | 1,13924862 | INTERFERON_GAMMA_RESPONSE |
| LY86 | 0,0017202 | 1,13283227 | ALLOGRAFT_REJECTION |
| FMO3 | 0,02479368 | 1,13224926 | XENOBIOTIC_METABOLISM |
| IL2RG | 0,00788397 | 1,13195042 | ALLOGRAFT_REJECTION |
| IL2RG | 0,00788397 | 1,13195042 | KRAS_SIGNALING_UP |
| IL1B | 0,00380637 | 1,13056937 | ALLOGRAFT_REJECTION |
| IL1B | 0,00380637 | 1,13056937 | INFLAMMATORY_RESPONSE |
| IL1B | 0,00380637 | 1,13056937 | KRAS_SIGNALING_UP |
| DOCK2 | 0,00225885 | 1,12866911 | KRAS_SIGNALING_UP |
| GNG2 | 0,00088285 | 1,12577458 | COMPLEMENT |
| PTGER3 | 0,01535873 | 1,12577434 | ESTROGEN_RESPONSE_LATE |
| CD14 | 0,00349658 | 1,12508891 | INFLAMMATORY_RESPONSE |
| MMD | 0,00349658 | 1,1247695 | KRAS_SIGNALING_UP |
| C1QC | 0,00269704 | 1,12466749 | COMPLEMENT |
| CD69 | 0,04643181 | 1,12449503 | INFLAMMATORY_RESPONSE |
| CD69 | 0,04643181 | 1,12449503 | INTERFERON_GAMMA_RESPONSE |
| TGFBI | 0,0017202 | 1,12443869 | EPITHELIAL_MESENCHYMAL_TRANSITION |
| VAMP8 | 0,00206457 | 1,12389502 | INTERFERON_GAMMA_RESPONSE |
| MFAP5 | 0,00574908 | 1,12335937 | EPITHELIAL_MESENCHYMAL_TRANSITION |
| PECAM1 | 0,00449973 | 1,12328755 | KRAS_SIGNALING_UP |
| PAPSS2 | 3,69E-05 | 1,12247303 | ESTROGEN_RESPONSE_EARLY |
| PAPSS2 | 3,69E-05 | 1,12247303 | ESTROGEN_RESPONSE_LATE |
| PAPSS2 | 3,69E-05 | 1,12247303 | XENOBIOTIC_METABOLISM |
| ITGA6 | 0,00246929 | 1,12157407 | IL2_STAT5_SIGNALING |
| CD74 | 0,0012996 | 1,11984599 | ALLOGRAFT_REJECTION |
| CD74 | 0,0012996 | 1,11984599 | INTERFERON_GAMMA_RESPONSE |
| IFI30 | 0,01430322 | 1,11729333 | INTERFERON_GAMMA_RESPONSE |
| IGSF6 | 0,00269704 | 1,117249 | ALLOGRAFT_REJECTION |
| IGSF6 | 0,00269704 | 1,117249 | ALLOGRAFT_REJECTION |
| IRF8 | 0,00156811 | 1,11420258 | ALLOGRAFT_REJECTION |
| IRF8 | 0,00156811 | 1,11420258 | IL2_STAT5_SIGNALING |
| IRF8 | 0,00156811 | 1,11420258 | INTERFERON_GAMMA_RESPONSE |
| IRF8 | 0,00156811 | 1,11420258 | KRAS_SIGNALING_UP |
| IRF8 | 0,00156811 | 1,11420258 | XENOBIOTIC_METABOLISM |
| PIK3CG | 0,00622855 | 1,11296928 | COMPLEMENT |
| CYBB | 0,00919046 | 1,1126108 | INFLAMMATORY_RESPONSE |
| IL10RA | 0,00188536 | 1,11223821 | IL2_STAT5_SIGNALING |
| IL10RA | 0,00188536 | 1,11223821 | INFLAMMATORY_RESPONSE |
| IL10RA | 0,00188536 | 1,11223821 | INTERFERON_GAMMA_RESPONSE |
| IL10RA | 0,00188536 | 1,11223821 | KRAS_SIGNALING_UP |
| CD48 | 0,00246929 | 1,11131759 | IL2_STAT5_SIGNALING |
| CD48 | 0,00246929 | 1,11131759 | INFLAMMATORY_RESPONSE |
| FCGR2B | 0,01237833 | 1,111235 | ALLOGRAFT_REJECTION |
| LYN | 0,0011815 | 1,11069642 | ALLOGRAFT_REJECTION |
| LYN | 0,0011815 | 1,11069642 | COMPLEMENT |
| LYN | 0,0011815 | 1,11069642 | INFLAMMATORY_RESPONSE |
| LYN | 0,0011815 | 1,11069642 | UV_RESPONSE_UP |
| SLIT3 | 3,69E-05 | 1,11057494 | EPITHELIAL_MESENCHYMAL_TRANSITION |
| MMP13 | 0,03013416 | 1,11018122 | COMPLEMENT |
| MMP13 | 0,03013416 | 1,11018122 | COMPLEMENT |
| INHBA | 0,0017202 | 1,10988479 | ALLOGRAFT_REJECTION |
| INHBA | 0,0017202 | 1,10988479 | INFLAMMATORY_RESPONSE |
| INHBA | 0,0017202 | 1,10988479 | KRAS_SIGNALING_UP |
| INHBA | 0,0017202 | 1,10988479 | EPITHELIAL_MESENCHYMAL_TRANSITION |
| C1QA | 0,00622855 | 1,1092448 | COMPLEMENT |
| EPB41L3 | 0,00079963 | 1,10890908 | KRAS_SIGNALING_UP |
| COL1A1 | 1,65E-05 | 1,10856655 | EPITHELIAL_MESENCHYMAL_TRANSITION |
| CD86 | 0,02479368 | 1,10815724 | ALLOGRAFT_REJECTION |
| CD86 | 0,02479368 | 1,10815724 | IL2_STAT5_SIGNALING |
| CD86 | 0,02479368 | 1,10815724 | INTERFERON_GAMMA_RESPONSE |
| PTAFR | 0,00349658 | 1,10773341 | INFLAMMATORY_RESPONSE |
| GZMA | 0,04926421 | 1,10723405 | ALLOGRAFT_REJECTION |
| GZMA | 0,04926421 | 1,10723405 | COMPLEMENT |
| GZMA | 0,04926421 | 1,10723405 | INTERFERON_GAMMA_RESPONSE |
| PRKCH | 0,0014282 | 1,10657231 | IL2_STAT5_SIGNALING |
| CD3G | 0,03013416 | 1,10567791 | ALLOGRAFT_REJECTION |
| CD4 | 0,0005907 | 1,10490765 | ALLOGRAFT_REJECTION |
| CPM | 0,01646281 | 1,10459509 | COMPLEMENT |
| KCNA3 | 0,01430322 | 1,10384594 | INFLAMMATORY_RESPONSE |
| ITGB2 | 0,0012996 | 1,10299189 | ALLOGRAFT_REJECTION |
| ITGB2 | 0,0012996 | 1,10299189 | KRAS_SIGNALING_UP |
| FARP1 | 6,96E-05 | 1,10145208 | ESTROGEN_RESPONSE_EARLY |
| FARP1 | 6,96E-05 | 1,10145208 | ESTROGEN_RESPONSE_LATE |
| LCP2 | 0,00156811 | 1,10078736 | ALLOGRAFT_REJECTION |
| LCP2 | 0,00156811 | 1,10078736 | COMPLEMENT |
| LCP2 | 0,00156811 | 1,10078736 | INFLAMMATORY_RESPONSE |
| LCP2 | 0,00156811 | 1,10078736 | INTERFERON_GAMMA_RESPONSE |
| POSTN | 0,01430322 | 1,10029091 | ANGIOGENESIS |
| POSTN | 0,01430322 | 1,10029091 | EPITHELIAL_MESENCHYMAL_TRANSITION |
| SERPINA1 | 0,00188536 | 1,09944939 | COMPLEMENT |
| SERPINA1 | 0,00188536 | 1,09944939 | COMPLEMENT |
| SERPINA1 | 0,00188536 | 1,09944939 | ESTROGEN_RESPONSE_LATE |
| SERPINA1 | 0,00188536 | 1,09944939 | ESTROGEN_RESPONSE_LATE |
| HLA-DMA | 0,00449973 | 1,09907403 | ALLOGRAFT_REJECTION |
| HLA-DMA | 0,00449973 | 1,09907403 | ALLOGRAFT_REJECTION |
| HLA-DMA | 0,00449973 | 1,09907403 | ALLOGRAFT_REJECTION |
| HLA-DMA | 0,00449973 | 1,09907403 | ALLOGRAFT_REJECTION |
| HLA-DMA | 0,00449973 | 1,09907403 | ALLOGRAFT_REJECTION |
| HLA-DMA | 0,00449973 | 1,09907403 | ALLOGRAFT_REJECTION |
| HLA-DMA | 0,00449973 | 1,09907403 | ALLOGRAFT_REJECTION |
| HLA-DMA | 0,00449973 | 1,09907403 | ALLOGRAFT_REJECTION |
| HLA-DMA | 0,00449973 | 1,09907403 | INTERFERON_GAMMA_RESPONSE |
| HLA-DMA | 0,00449973 | 1,09907403 | INTERFERON_GAMMA_RESPONSE |
| HLA-DMA | 0,00449973 | 1,09907403 | INTERFERON_GAMMA_RESPONSE |
| HLA-DMA | 0,00449973 | 1,09907403 | INTERFERON_GAMMA_RESPONSE |
| HLA-DMA | 0,00449973 | 1,09907403 | INTERFERON_GAMMA_RESPONSE |
| HLA-DMA | 0,00449973 | 1,09907403 | INTERFERON_GAMMA_RESPONSE |
| HLA-DMA | 0,00449973 | 1,09907403 | INTERFERON_GAMMA_RESPONSE |
| HLA-DMA | 0,00449973 | 1,09907403 | INTERFERON_GAMMA_RESPONSE |
| COL12A1 | 0,00107314 | 1,0985889 | EPITHELIAL_MESENCHYMAL_TRANSITION |
| IFITM1 | 0,04116831 | 1,09842092 | INFLAMMATORY_RESPONSE |
| CTSC | 0,00574908 | 1,09811768 | COMPLEMENT |
| CA12 | 0,02320174 | 1,097309 | ESTROGEN_RESPONSE_EARLY |
| CA12 | 0,02320174 | 1,097309 | ESTROGEN_RESPONSE_LATE |
| MXRA5 | 0,00622855 | 1,09651934 | EPITHELIAL_MESENCHYMAL_TRANSITION |
| TLR8 | 0,0029433 | 1,09427979 | KRAS_SIGNALING_UP |
| OAS2 | 0,01535873 | 1,09370726 | INTERFERON_GAMMA_RESPONSE |
| CD96 | 0,00729386 | 1,09365327 | ALLOGRAFT_REJECTION |
| DOCK10 | 0,00206457 | 1,0932077 | COMPLEMENT |
| ITGAM | 0,00488654 | 1,09239557 | COMPLEMENT |
| CD2 | 0,02169734 | 1,09193926 | ALLOGRAFT_REJECTION |
| MX2 | 0,00574908 | 1,09123856 | INTERFERON_GAMMA_RESPONSE |
| CLEC4A | 0,01430322 | 1,09106844 | KRAS_SIGNALING_UP |
| C2 | 0,02320174 | 1,09104333 | ALLOGRAFT_REJECTION |
| C2 | 0,02320174 | 1,09104333 | ALLOGRAFT_REJECTION |
| C2 | 0,02320174 | 1,09104333 | ALLOGRAFT_REJECTION |
| C2 | 0,02320174 | 1,09104333 | ALLOGRAFT_REJECTION |
| C2 | 0,02320174 | 1,09104333 | ALLOGRAFT_REJECTION |
| C2 | 0,02320174 | 1,09104333 | ALLOGRAFT_REJECTION |
| C2 | 0,02320174 | 1,09104333 | ALLOGRAFT_REJECTION |
| C2 | 0,02320174 | 1,09104333 | COMPLEMENT |
| C2 | 0,02320174 | 1,09104333 | COMPLEMENT |
| C2 | 0,02320174 | 1,09104333 | COMPLEMENT |
| C2 | 0,02320174 | 1,09104333 | COMPLEMENT |
| C2 | 0,02320174 | 1,09104333 | COMPLEMENT |
| C2 | 0,02320174 | 1,09104333 | COMPLEMENT |
| C2 | 0,02320174 | 1,09104333 | COMPLEMENT |
| RASGRP1 | 0,00991168 | 1,09036502 | COMPLEMENT |
| RASGRP1 | 0,00991168 | 1,09036502 | ESTROGEN_RESPONSE_EARLY |
| RASGRP1 | 0,00991168 | 1,09036502 | INFLAMMATORY_RESPONSE |
| RASGRP1 | 0,00991168 | 1,09036502 | UV_RESPONSE_UP |
| TLR1 | 0,02027666 | 1,09026997 | ALLOGRAFT_REJECTION |
| TLR1 | 0,02027666 | 1,09026997 | INFLAMMATORY_RESPONSE |
| EPSTI1 | 0,01430322 | 1,09000473 | INTERFERON_GAMMA_RESPONSE |
| SAMD9L | 0,00674278 | 1,08966115 | INTERFERON_GAMMA_RESPONSE |
| PRDM1 | 0,00349658 | 1,0892303 | KRAS_SIGNALING_UP |
| TMEM176A | 0,00788397 | 1,08894784 | KRAS_SIGNALING_UP |
| NID2 | 0,03872779 | 1,08740263 | EPITHELIAL_MESENCHYMAL_TRANSITION |
| TOP2A | 0,01648049 | 1,08709808 | ESTROGEN_RESPONSE_LATE |
| VEGFA | 0,02479368 | 1,08677222 | ANGIOGENESIS |
| VEGFA | 0,02479368 | 1,08677222 | EPITHELIAL_MESENCHYMAL_TRANSITION |
| F2R | 0,0012996 | 1,08506081 | ALLOGRAFT_REJECTION |
| FPR1 | 0,0012996 | 1,08458808 | INFLAMMATORY_RESPONSE |
| FPR1 | 0,0012996 | 1,08458808 | INTERFERON_GAMMA_RESPONSE |
| PELI1 | 0,00380637 | 1,08426533 | INTERFERON_GAMMA_RESPONSE |
| HLA-DRA | 0,00729386 | 1,0840529 | ALLOGRAFT_REJECTION |
| HLA-DRA | 0,00729386 | 1,0840529 | ALLOGRAFT_REJECTION |
| HLA-DRA | 0,00729386 | 1,0840529 | ALLOGRAFT_REJECTION |
| HLA-DRA | 0,00729386 | 1,0840529 | ALLOGRAFT_REJECTION |
| HLA-DRA | 0,00729386 | 1,0840529 | ALLOGRAFT_REJECTION |
| HLA-DRA | 0,00729386 | 1,0840529 | ALLOGRAFT_REJECTION |
| HLA-DRA | 0,00729386 | 1,0840529 | ALLOGRAFT_REJECTION |
| HLA-DRA | 0,00729386 | 1,0840529 | ALLOGRAFT_REJECTION |
| P2RX7 | 0,00788397 | 1,08329616 | INFLAMMATORY_RESPONSE |
| CD37 | 0,02320174 | 1,08323348 | KRAS_SIGNALING_UP |
| LGALS3BP | 1,65E-05 | 1,08308553 | INTERFERON_GAMMA_RESPONSE |
| C5AR1 | 0,00053293 | 1,08040197 | INFLAMMATORY_RESPONSE |
| LAMA3 | 0,00919046 | 1,08025583 | EPITHELIAL_MESENCHYMAL_TRANSITION |
| FAP | 0,00622855 | 1,08011357 | EPITHELIAL_MESENCHYMAL_TRANSITION |
| CD80 | 0,01535873 | 1,0782294 | ALLOGRAFT_REJECTION |
| CDH6 | 0,00088285 | 1,07784695 | EPITHELIAL_MESENCHYMAL_TRANSITION |
| RNF213 | 0,000389 | 1,07638323 | INTERFERON_GAMMA_RESPONSE |
| THBS1 | 0,0106816 | 1,07623555 | EPITHELIAL_MESENCHYMAL_TRANSITION |
| IGF1 | 0,00991168 | 1,07567906 | XENOBIOTIC_METABOLISM |
| SAMHD1 | 0,00530238 | 1,07559463 | INTERFERON_GAMMA_RESPONSE |
| IL4R | 0,0002813 | 1,07526706 | ALLOGRAFT_REJECTION |
| IL4R | 0,0002813 | 1,07526706 | IL2_STAT5_SIGNALING |
| IL4R | 0,0002813 | 1,07526706 | INFLAMMATORY_RESPONSE |
| IL4R | 0,0002813 | 1,07526706 | INTERFERON_GAMMA_RESPONSE |
| ATP2B1 | 0,02027666 | 1,07428818 | INFLAMMATORY_RESPONSE |
| COL4A1 | 0,00017984 | 1,07390517 | EPITHELIAL_MESENCHYMAL_TRANSITION |
| ENG | 0,00097381 | 1,07274738 | KRAS_SIGNALING_UP |
| IGFBP3 | 0,01331078 | 1,07269547 | KRAS_SIGNALING_UP |
| IGFBP3 | 0,01331078 | 1,07269547 | EPITHELIAL_MESENCHYMAL_TRANSITION |
| ECM1 | 0,00206457 | 1,0724076 | EPITHELIAL_MESENCHYMAL_TRANSITION |
| ECM1 | 0,00206457 | 1,0724076 | IL2_STAT5_SIGNALING |
| SNX10 | 0,02320174 | 1,07233426 | ESTROGEN_RESPONSE_LATE |
| HCLS1 | 0,00449973 | 1,07187674 | ALLOGRAFT_REJECTION |
| ITGAL | 0,00188536 | 1,07185287 | ALLOGRAFT_REJECTION |
| DOCK4 | 0,02027666 | 1,07087238 | COMPLEMENT |
| ELF4 | 2,48E-05 | 1,07072869 | ALLOGRAFT_REJECTION |
| NRP1 | 0,01893601 | 1,07065968 | ANGIOGENESIS |
| NRP1 | 0,01893601 | 1,07065968 | IL2_STAT5_SIGNALING |
| NRP1 | 0,01893601 | 1,07065968 | KRAS_SIGNALING_UP |
| IKZF1 | 0,00380637 | 1,07037314 | KRAS_SIGNALING_UP |
| ARRB2 | 0,00048033 | 1,07002918 | UV_RESPONSE_UP |
| FBN1 | 6,96E-05 | 1,06969201 | EPITHELIAL_MESENCHYMAL_TRANSITION |
| OPN3 | 0,00206457 | 1,06839368 | ESTROGEN_RESPONSE_EARLY |
| OPN3 | 0,00206457 | 1,06839368 | ESTROGEN_RESPONSE_LATE |
| PLAU | 0,0029433 | 1,06793818 | KRAS_SIGNALING_UP |
| SGK1 | 0,01430322 | 1,06783108 | ESTROGEN_RESPONSE_LATE |
| GSR | 1,89E-05 | 1,06697564 | XENOBIOTIC_METABOLISM |
| IL1R1 | 0,03640861 | 1,06682913 | INFLAMMATORY_RESPONSE |
| IL1R1 | 0,03640861 | 1,06682913 | XENOBIOTIC_METABOLISM |
| SPRY4 | 0,01150289 | 1,06663778 | IL2_STAT5_SIGNALING |
| ST6GAL1 | 0,00020146 | 1,06655364 | KRAS_SIGNALING_UP |
| MYO1E | 1,43E-05 | 1,06640938 | IL2_STAT5_SIGNALING |
| HIF1A | 0,00729386 | 1,06499404 | ALLOGRAFT_REJECTION |
| HIF1A | 0,00729386 | 1,06499404 | INFLAMMATORY_RESPONSE |
| HIF1A | 0,00729386 | 1,06499404 | INTERFERON_GAMMA_RESPONSE |
| IDH2 | 2,84E-05 | 1,06453766 | ESTROGEN_RESPONSE_LATE |
| HMOX1 | 0,01237833 | 1,06430231 | UV_RESPONSE_UP |
| HMOX1 | 0,01237833 | 1,06430231 | XENOBIOTIC_METABOLISM |
| RHOH | 0,00097381 | 1,06375241 | IL2_STAT5_SIGNALING |
| PSMB8 | 0,01767178 | 1,0636428 | INTERFERON_GAMMA_RESPONSE |
| PSMB8 | 0,01767178 | 1,0636428 | INTERFERON_GAMMA_RESPONSE |
| PSMB8 | 0,01767178 | 1,0636428 | INTERFERON_GAMMA_RESPONSE |
| PSMB8 | 0,01767178 | 1,0636428 | INTERFERON_GAMMA_RESPONSE |
| PSMB8 | 0,01767178 | 1,0636428 | INTERFERON_GAMMA_RESPONSE |
| PSMB8 | 0,01767178 | 1,0636428 | INTERFERON_GAMMA_RESPONSE |
| PSMB8 | 0,01767178 | 1,0636428 | INTERFERON_GAMMA_RESPONSE |
| PSMB8 | 0,01767178 | 1,0636428 | INTERFERON_GAMMA_RESPONSE |
| PSMB8 | 0,01767178 | 1,0636428 | KRAS_SIGNALING_UP |
| PSMB8 | 0,01767178 | 1,0636428 | KRAS_SIGNALING_UP |
| PSMB8 | 0,01767178 | 1,0636428 | KRAS_SIGNALING_UP |
| PSMB8 | 0,01767178 | 1,0636428 | KRAS_SIGNALING_UP |
| PSMB8 | 0,01767178 | 1,0636428 | KRAS_SIGNALING_UP |
| PSMB8 | 0,01767178 | 1,0636428 | KRAS_SIGNALING_UP |
| PSMB8 | 0,01767178 | 1,0636428 | KRAS_SIGNALING_UP |
| PSMB8 | 0,01767178 | 1,0636428 | KRAS_SIGNALING_UP |
| SLC6A6 | 0,01150289 | 1,06338042 | XENOBIOTIC_METABOLISM |
| PTGER4 | 0,00729386 | 1,06315862 | INFLAMMATORY_RESPONSE |
| CIITA | 0,00574908 | 1,06293682 | INTERFERON_GAMMA_RESPONSE |
| LGMN | 0,00246929 | 1,06240925 | COMPLEMENT |
| SLC46A3 | 0,0321162 | 1,06233654 | XENOBIOTIC_METABOLISM |
| NRIP1 | 0,02479368 | 1,06207517 | ESTROGEN_RESPONSE_EARLY |
| NRIP1 | 0,02479368 | 1,06207517 | ESTROGEN_RESPONSE_LATE |
| PPP1R2 | 0,01237833 | 1,06200704 | UV_RESPONSE_UP |
| DNMBP | 0,00017984 | 1,06186929 | KRAS_SIGNALING_UP |
| PTPN6 | 0,01767178 | 1,06129433 | ALLOGRAFT_REJECTION |
| PTPN6 | 0,01767178 | 1,06129433 | ESTROGEN_RESPONSE_LATE |
| PTPN6 | 0,01767178 | 1,06129433 | INTERFERON_GAMMA_RESPONSE |
| IFNAR2 | 0,00225885 | 1,0606612 | ALLOGRAFT_REJECTION |
| IFNAR2 | 0,00225885 | 1,0606612 | INTERFERON_GAMMA_RESPONSE |
| LOXL2 | 0,01150289 | 1,06062648 | EPITHELIAL_MESENCHYMAL_TRANSITION |
| ITGA2 | 0,03872779 | 1,06037215 | KRAS_SIGNALING_UP |
| ITGA2 | 0,03872779 | 1,06037215 | EPITHELIAL_MESENCHYMAL_TRANSITION |
| LIPA | 0,0321162 | 1,06016442 | COMPLEMENT |
| NIN | 0,0011815 | 1,06005899 | KRAS_SIGNALING_UP |
| SLC24A3 | 0,0011815 | 1,06004467 | ESTROGEN_RESPONSE_EARLY |
| SLC24A3 | 0,0011815 | 1,06004467 | ESTROGEN_RESPONSE_LATE |
| STAB1 | 0,02169734 | 1,05977786 | ALLOGRAFT_REJECTION |
| STAB1 | 0,02169734 | 1,05977786 | INFLAMMATORY_RESPONSE |
| PLAUR | 0,00156811 | 1,05920928 | EPITHELIAL_MESENCHYMAL_TRANSITION |
| PLAUR | 0,00156811 | 1,05920928 | COMPLEMENT |
| PLAUR | 0,00156811 | 1,05920928 | INFLAMMATORY_RESPONSE |
| PLAUR | 0,00156811 | 1,05920928 | KRAS_SIGNALING_UP |
| MMP14 | 0,0011815 | 1,05828556 | EPITHELIAL_MESENCHYMAL_TRANSITION |
| MMP14 | 0,0011815 | 1,05828556 | COMPLEMENT |
| MMP14 | 0,0011815 | 1,05828556 | INFLAMMATORY_RESPONSE |
| MMP14 | 0,0011815 | 1,05828556 | UV_RESPONSE_UP |
| PCOLCE | 0,03420622 | 1,05637329 | EPITHELIAL_MESENCHYMAL_TRANSITION |
| CBL | 0,000389 | 1,05575321 | KRAS_SIGNALING_UP |
| MYOF | 0,00053293 | 1,05523188 | ESTROGEN_RESPONSE_EARLY |
| MYOF | 0,00053293 | 1,05523188 | ESTROGEN_RESPONSE_LATE |
| ETS1 | 0,00246929 | 1,05520891 | ALLOGRAFT_REJECTION |
| ETS1 | 0,00246929 | 1,05520891 | KRAS_SIGNALING_UP |
| COL4A2 | 0,00043248 | 1,05508616 | EPITHELIAL_MESENCHYMAL_TRANSITION |
| COL4A2 | 0,00043248 | 1,05508616 | COMPLEMENT |
| THBS2 | 0,01430322 | 1,05485362 | EPITHELIAL_MESENCHYMAL_TRANSITION |
| FYN | 0,04116831 | 1,0540978 | COMPLEMENT |
| STAT2 | 0,00043248 | 1,05383914 | INTERFERON_GAMMA_RESPONSE |
| APOL6 | 0,02479368 | 1,05329638 | INTERFERON_GAMMA_RESPONSE |
| TNFAIP2 | 0,01648049 | 1,05303799 | INTERFERON_GAMMA_RESPONSE |
| ZNFX1 | 0,00043248 | 1,05297675 | INTERFERON_GAMMA_RESPONSE |
| PLEK2 | 0,04643181 | 1,05262502 | KRAS_SIGNALING_UP |
| CYB5A | 0,01430322 | 1,05246799 | XENOBIOTIC_METABOLISM |
| COL3A1 | 0,00574908 | 1,05180907 | EPITHELIAL_MESENCHYMAL_TRANSITION |
| COL3A1 | 0,00574908 | 1,05180907 | ANGIOGENESIS |
| PRSS23 | 0,00788397 | 1,05165848 | ESTROGEN_RESPONSE_EARLY |
| PRSS23 | 0,00788397 | 1,05165848 | ESTROGEN_RESPONSE_LATE |
| IGF1R | 0,00919046 | 1,05141709 | ESTROGEN_RESPONSE_EARLY |
| IGF1R | 0,00919046 | 1,05141709 | IL2_STAT5_SIGNALING |
| COL5A1 | 0,00246929 | 1,0510298 | EPITHELIAL_MESENCHYMAL_TRANSITION |
| ECM2 | 0,00729386 | 1,05092362 | EPITHELIAL_MESENCHYMAL_TRANSITION |
| JUP | 2,01E-06 | 1,05080824 | KRAS_SIGNALING_UP |
| JUP | 2,01E-06 | 1,05080824 | XENOBIOTIC_METABOLISM |
| GPR18 | 0,02169734 | 1,05075502 | INTERFERON_GAMMA_RESPONSE |
| ITGA5 | 0,00107314 | 1,05006536 | EPITHELIAL_MESENCHYMAL_TRANSITION |
| ITGA5 | 0,00107314 | 1,05006536 | INFLAMMATORY_RESPONSE |
| GPR132 | 0,00156811 | 1,0499923 | INFLAMMATORY_RESPONSE |
| STAT4 | 0,03013416 | 1,04921455 | ALLOGRAFT_REJECTION |
| STAT4 | 0,03013416 | 1,04921455 | INTERFERON_GAMMA_RESPONSE |
| DLC1 | 0,00674278 | 1,04918258 | ESTROGEN_RESPONSE_EARLY |
| DLC1 | 0,00674278 | 1,04918258 | ESTROGEN_RESPONSE_EARLY |
| SP110 | 0,0106816 | 1,04896483 | INTERFERON_GAMMA_RESPONSE |
| TIAM1 | 0,02320174 | 1,0489175 | ESTROGEN_RESPONSE_EARLY |
| TIAM1 | 0,02320174 | 1,0489175 | ESTROGEN_RESPONSE_LATE |
| TIAM1 | 0,02320174 | 1,0489175 | IL2_STAT5_SIGNALING |
| IRF1 | 0,0321162 | 1,04891311 | COMPLEMENT |
| IRF1 | 0,0321162 | 1,04891311 | INFLAMMATORY_RESPONSE |
| IRF1 | 0,0321162 | 1,04891311 | INTERFERON_GAMMA_RESPONSE |
| IRF1 | 0,0321162 | 1,04891311 | UV_RESPONSE_UP |
| RNF144B | 0,0321162 | 1,04856 | INFLAMMATORY_RESPONSE |
| ETV5 | 0,00788397 | 1,04854275 | KRAS_SIGNALING_UP |
| CDH11 | 0,00206457 | 1,04843016 | EPITHELIAL_MESENCHYMAL_TRANSITION |
| NLRP3 | 0,00851538 | 1,04797375 | ALLOGRAFT_REJECTION |
| NLRP3 | 0,00851538 | 1,04797375 | INFLAMMATORY_RESPONSE |
| INPP5F | 0,01893601 | 1,04766835 | ESTROGEN_RESPONSE_EARLY |
| FAS | 0,01331078 | 1,04760249 | EPITHELIAL_MESENCHYMAL_TRANSITION |
| FAS | 0,01331078 | 1,04760249 | ALLOGRAFT_REJECTION |
| FAS | 0,01331078 | 1,04760249 | INTERFERON_GAMMA_RESPONSE |
| FAS | 0,01331078 | 1,04760249 | XENOBIOTIC_METABOLISM |
| TGFB1 | 0,00349658 | 1,04749528 | EPITHELIAL_MESENCHYMAL_TRANSITION |
| TGFB1 | 0,00349658 | 1,04749528 | ALLOGRAFT_REJECTION |
| RHOG | 0,0014282 | 1,04709508 | COMPLEMENT |
| RHOG | 0,0014282 | 1,04709508 | INFLAMMATORY_RESPONSE |
| MTMR10 | 0,00246929 | 1,0469593 | KRAS_SIGNALING_UP |
| MTMR10 | 0,00246929 | 1,0469593 | KRAS_SIGNALING_UP |
| COL6A3 | 0,01767178 | 1,04680198 | EPITHELIAL_MESENCHYMAL_TRANSITION |
| SOCS3 | 0,04373476 | 1,04634471 | INTERFERON_GAMMA_RESPONSE |
| SH3BGRL2 | 0,03872779 | 1,04582511 | IL2_STAT5_SIGNALING |
| TNFRSF1B | 0,02027666 | 1,04581101 | IL2_STAT5_SIGNALING |
| TNFRSF1B | 0,02027666 | 1,04581101 | INFLAMMATORY_RESPONSE |
| TNFRSF1B | 0,02027666 | 1,04581101 | KRAS_SIGNALING_UP |
| CD83 | 0,00729386 | 1,04472718 | IL2_STAT5_SIGNALING |
| IL16 | 0,01237833 | 1,04352745 | ALLOGRAFT_REJECTION |
| USP8 | 0,01893601 | 1,04323665 | COMPLEMENT |
| AXL | 0,0017202 | 1,0430844 | INFLAMMATORY_RESPONSE |
| HLA-F | 0,02320174 | 1,04295148 | UV_RESPONSE_UP |
| HLA-F | 0,02320174 | 1,04295148 | UV_RESPONSE_UP |
| HLA-F | 0,02320174 | 1,04295148 | UV_RESPONSE_UP |
| HLA-F | 0,02320174 | 1,04295148 | UV_RESPONSE_UP |
| HLA-F | 0,02320174 | 1,04295148 | UV_RESPONSE_UP |
| HLA-F | 0,02320174 | 1,04295148 | UV_RESPONSE_UP |
| HLA-F | 0,02320174 | 1,04295148 | UV_RESPONSE_UP |
| CAPN3 | 0,0011815 | 1,04294599 | IL2_STAT5_SIGNALING |
| PTS | 0,01535873 | 1,04281556 | XENOBIOTIC_METABOLISM |
| DDX21 | 0,02169734 | 1,0424737 | UV_RESPONSE_UP |
| SERPINH1 | 0,00022542 | 1,04244442 | EPITHELIAL_MESENCHYMAL_TRANSITION |
| IL10 | 0,02825584 | 1,04243005 | ALLOGRAFT_REJECTION |
| IL10 | 0,02825584 | 1,04243005 | IL2_STAT5_SIGNALING |
| IL10 | 0,02825584 | 1,04243005 | INFLAMMATORY_RESPONSE |
| CXCR6 | 0,0321162 | 1,04219506 | INFLAMMATORY_RESPONSE |
| OAS3 | 0,0321162 | 1,04208243 | INTERFERON_GAMMA_RESPONSE |
| ADAM17 | 0,0321162 | 1,0417324 | KRAS_SIGNALING_UP |
| PLOD1 | 0,00574908 | 1,04144263 | EPITHELIAL_MESENCHYMAL_TRANSITION |
| SHMT2 | 0,00674278 | 1,04125977 | XENOBIOTIC_METABOLISM |
| TNFRSF1A | 0,000389 | 1,04005227 | XENOBIOTIC_METABOLISM |
| SLC31A2 | 0,02320174 | 1,03990827 | INFLAMMATORY_RESPONSE |
| ADAM12 | 0,01430322 | 1,03988828 | EPITHELIAL_MESENCHYMAL_TRANSITION |
| CSK | 0,00088285 | 1,03980157 | ALLOGRAFT_REJECTION |
| TAPBP | 0,00414023 | 1,03977364 | ALLOGRAFT_REJECTION |
| TAPBP | 0,00414023 | 1,03977364 | ALLOGRAFT_REJECTION |
| TAPBP | 0,00414023 | 1,03977364 | ALLOGRAFT_REJECTION |
| TAPBP | 0,00414023 | 1,03977364 | ALLOGRAFT_REJECTION |
| TAPBP | 0,00414023 | 1,03977364 | ALLOGRAFT_REJECTION |
| TAPBP | 0,00414023 | 1,03977364 | INFLAMMATORY_RESPONSE |
| TAPBP | 0,00414023 | 1,03977364 | INFLAMMATORY_RESPONSE |
| TAPBP | 0,00414023 | 1,03977364 | INFLAMMATORY_RESPONSE |
| TAPBP | 0,00414023 | 1,03977364 | INFLAMMATORY_RESPONSE |
| TAPBP | 0,00414023 | 1,03977364 | INFLAMMATORY_RESPONSE |
| TAPBP | 0,00414023 | 1,03977364 | INTERFERON_GAMMA_RESPONSE |
| TAPBP | 0,00414023 | 1,03977364 | INTERFERON_GAMMA_RESPONSE |
| TAPBP | 0,00414023 | 1,03977364 | INTERFERON_GAMMA_RESPONSE |
| TAPBP | 0,00414023 | 1,03977364 | INTERFERON_GAMMA_RESPONSE |
| TAPBP | 0,00414023 | 1,03977364 | INTERFERON_GAMMA_RESPONSE |
| PML | 0,00097381 | 1,03904377 | INTERFERON_GAMMA_RESPONSE |
| PPIF | 0,00729386 | 1,03886285 | ESTROGEN_RESPONSE_EARLY |
| PPIF | 0,00729386 | 1,03886285 | ESTROGEN_RESPONSE_LATE |
| PPIF | 0,00729386 | 1,03886285 | UV_RESPONSE_UP |
| NPC1 | 0,00320937 | 1,03844173 | XENOBIOTIC_METABOLISM |
| LRP1 | 0,00225885 | 1,0382088 | EPITHELIAL_MESENCHYMAL_TRANSITION |
| LRP1 | 0,00225885 | 1,0382088 | COMPLEMENT |
| SAT1 | 0,04373476 | 1,03802535 | EPITHELIAL_MESENCHYMAL_TRANSITION |
| TNFAIP3 | 0,02825584 | 1,03797485 | EPITHELIAL_MESENCHYMAL_TRANSITION |
| TNFAIP3 | 0,02825584 | 1,03797485 | COMPLEMENT |
| TNFAIP3 | 0,02825584 | 1,03797485 | INTERFERON_GAMMA_RESPONSE |
| TNFAIP3 | 0,02825584 | 1,03797485 | KRAS_SIGNALING_UP |
| NLRC5 | 0,02169734 | 1,03764343 | INTERFERON_GAMMA_RESPONSE |
| RABEP1 | 0,02479368 | 1,03719281 | ESTROGEN_RESPONSE_LATE |
| PTPRE | 0,03013416 | 1,03703506 | INFLAMMATORY_RESPONSE |
| MED13L | 0,00851538 | 1,03683955 | ESTROGEN_RESPONSE_EARLY |
| PRF1 | 0,03013416 | 1,03622968 | ALLOGRAFT_REJECTION |
| ACVR1B | 0,00072355 | 1,03596206 | INFLAMMATORY_RESPONSE |
| CRLF1 | 0,01535873 | 1,03584508 | EPITHELIAL_MESENCHYMAL_TRANSITION |
| BTG1 | 0,02027666 | 1,03568282 | INTERFERON_GAMMA_RESPONSE |
| BTG1 | 0,02027666 | 1,03568282 | UV_RESPONSE_UP |
| COL1A2 | 0,00349658 | 1,03535705 | EPITHELIAL_MESENCHYMAL_TRANSITION |
| NCOA3 | 0,04926421 | 1,03440485 | IL2_STAT5_SIGNALING |
| NCOA3 | 0,04926421 | 1,03440485 | INTERFERON_GAMMA_RESPONSE |
| MAP3K8 | 0,01767178 | 1,03421652 | IL2_STAT5_SIGNALING |
| TAP1 | 0,02320174 | 1,03385929 | ALLOGRAFT_REJECTION |
| TAP1 | 0,02320174 | 1,03385929 | ALLOGRAFT_REJECTION |
| TAP1 | 0,02320174 | 1,03385929 | ALLOGRAFT_REJECTION |
| TAP1 | 0,02320174 | 1,03385929 | ALLOGRAFT_REJECTION |
| TAP1 | 0,02320174 | 1,03385929 | ALLOGRAFT_REJECTION |
| TAP1 | 0,02320174 | 1,03385929 | ALLOGRAFT_REJECTION |
| TAP1 | 0,02320174 | 1,03385929 | ALLOGRAFT_REJECTION |
| TAP1 | 0,02320174 | 1,03385929 | ALLOGRAFT_REJECTION |
| TAP1 | 0,02320174 | 1,03385929 | INTERFERON_GAMMA_RESPONSE |
| TAP1 | 0,02320174 | 1,03385929 | INTERFERON_GAMMA_RESPONSE |
| TAP1 | 0,02320174 | 1,03385929 | INTERFERON_GAMMA_RESPONSE |
| TAP1 | 0,02320174 | 1,03385929 | INTERFERON_GAMMA_RESPONSE |
| TAP1 | 0,02320174 | 1,03385929 | INTERFERON_GAMMA_RESPONSE |
| TAP1 | 0,02320174 | 1,03385929 | INTERFERON_GAMMA_RESPONSE |
| TAP1 | 0,02320174 | 1,03385929 | INTERFERON_GAMMA_RESPONSE |
| TAP1 | 0,02320174 | 1,03385929 | INTERFERON_GAMMA_RESPONSE |
| TAP1 | 0,02320174 | 1,03385929 | UV_RESPONSE_UP |
| TAP1 | 0,02320174 | 1,03385929 | UV_RESPONSE_UP |
| TAP1 | 0,02320174 | 1,03385929 | UV_RESPONSE_UP |
| TAP1 | 0,02320174 | 1,03385929 | UV_RESPONSE_UP |
| TAP1 | 0,02320174 | 1,03385929 | UV_RESPONSE_UP |
| TAP1 | 0,02320174 | 1,03385929 | UV_RESPONSE_UP |
| TAP1 | 0,02320174 | 1,03385929 | UV_RESPONSE_UP |
| TAP1 | 0,02320174 | 1,03385929 | UV_RESPONSE_UP |
| COL5A2 | 0,02479368 | 1,0337041 | EPITHELIAL_MESENCHYMAL_TRANSITION |
| COL5A2 | 0,02479368 | 1,0337041 | ANGIOGENESIS |
| ACP2 | 0,00991168 | 1,03367222 | XENOBIOTIC_METABOLISM |
| EHD1 | 0,00530238 | 1,0336001 | COMPLEMENT |
| XPNPEP1 | 0,04643181 | 1,03312052 | COMPLEMENT |
| DENND5A | 0,00991168 | 1,0328785 | IL2_STAT5_SIGNALING |
| NDRG1 | 0,00349658 | 1,0324893 | IL2_STAT5_SIGNALING |
| SCUBE2 | 0,04856821 | 1,03232158 | ESTROGEN_RESPONSE_LATE |
| IGF2R | 0,01893601 | 1,03222908 | IL2_STAT5_SIGNALING |
| IL18BP | 0,01237833 | 1,03213792 | INTERFERON_GAMMA_RESPONSE |
| ITGBL1 | 0,00488654 | 1,03210152 | KRAS_SIGNALING_UP |
| CHKA | 0,04373476 | 1,03190089 | UV_RESPONSE_UP |
| TPM1 | 0,0017202 | 1,03159226 | EPITHELIAL_MESENCHYMAL_TRANSITION |
| SLC1A1 | 0,02169734 | 1,03153222 | ESTROGEN_RESPONSE_EARLY |
| RARA | 0,01535873 | 1,03133054 | ESTROGEN_RESPONSE_EARLY |
| NUP93 | 0,00088285 | 1,03058265 | INTERFERON_GAMMA_RESPONSE |
| HSPA5 | 0,02825584 | 1,03057619 | COMPLEMENT |
| VCAN | 0,01237833 | 1,02958797 | EPITHELIAL_MESENCHYMAL_TRANSITION |
| VCAN | 0,01237833 | 1,02958797 | ANGIOGENESIS |
| TRAF1 | 0,03872779 | 1,0295674 | IL2_STAT5_SIGNALING |
| TRAF1 | 0,03872779 | 1,0295674 | KRAS_SIGNALING_UP |
| LAT2 | 0,03013416 | 1,02933117 | KRAS_SIGNALING_UP |
| CALD1 | 0,01893601 | 1,0292881 | EPITHELIAL_MESENCHYMAL_TRANSITION |
| FURIN | 0,00034953 | 1,02876593 | IL2_STAT5_SIGNALING |
| FURIN | 0,00034953 | 1,02876593 | UV_RESPONSE_UP |
| NXF1 | 0,04926421 | 1,02869349 | UV_RESPONSE_UP |
| PRKCD | 0,00991168 | 1,0286326 | COMPLEMENT |
| PRKCD | 0,00991168 | 1,0286326 | UV_RESPONSE_UP |
| BID | 0,00674278 | 1,02861724 | UV_RESPONSE_UP |
| PFKP | 0,03420622 | 1,02836577 | INTERFERON_GAMMA_RESPONSE |
| BCL2L1 | 0,00206457 | 1,02835809 | IL2_STAT5_SIGNALING |
| SLC11A2 | 0,00919046 | 1,02827076 | INFLAMMATORY_RESPONSE |
| COL6A1 | 0,04926421 | 1,02797782 | IL2_STAT5_SIGNALING |
| CTSD | 0,00574908 | 1,0275826 | COMPLEMENT |
| SH2B3 | 0,00729386 | 1,02724355 | COMPLEMENT |
| TIMP2 | 0,00851538 | 1,02706738 | COMPLEMENT |
| THBD | 0,04373476 | 1,02702146 | ANGIOGENESIS |
| P2RX4 | 0,01767178 | 1,02688684 | IL2_STAT5_SIGNALING |
| P2RX4 | 0,01767178 | 1,02688684 | INFLAMMATORY_RESPONSE |
| ELN | 0,04373476 | 1,02673626 | EPITHELIAL_MESENCHYMAL_TRANSITION |
| SLC7A1 | 0,01767178 | 1,0265803 | INFLAMMATORY_RESPONSE |
| MARK2 | 0,00729386 | 1,02654935 | UV_RESPONSE_UP |
| MVP | 0,00320937 | 1,02620389 | INTERFERON_GAMMA_RESPONSE |
| APP | 0,008242 | 1,02561809 | ANGIOGENESIS |
| CNDP2 | 0,02320174 | 1,02523636 | XENOBIOTIC_METABOLISM |
| STAT3 | 0,01648049 | 1,02487089 | INTERFERON_GAMMA_RESPONSE |
| COMT | 0,01237833 | 1,02450905 | XENOBIOTIC_METABOLISM |
| VAV2 | 0,03640861 | 1,02436986 | ANGIOGENESIS |
| CYFIP1 | 0,03640861 | 1,02432801 | IL2_STAT5_SIGNALING |
| CYFIP1 | 0,03640861 | 1,02432801 | IL2_STAT5_SIGNALING |
| CYFIP1 | 0,03640861 | 1,02432801 | IL2_STAT5_SIGNALING |
| PTPN1 | 0,00043248 | 1,02419808 | INTERFERON_GAMMA_RESPONSE |
| MUC1 | 0,02647704 | 1,02415004 | ESTROGEN_RESPONSE_EARLY |
| MUC1 | 0,02647704 | 1,02415004 | IL2_STAT5_SIGNALING |
| PLOD3 | 0,01535873 | 1,02403996 | EPITHELIAL_MESENCHYMAL_TRANSITION |
| CTSZ | 0,00188536 | 1,02373448 | IL2_STAT5_SIGNALING |
| BMP1 | 0,03640861 | 1,02364137 | EPITHELIAL_MESENCHYMAL_TRANSITION |
| RAB31 | 0,04373476 | 1,02347974 | ESTROGEN_RESPONSE_EARLY |
| RAB31 | 0,04373476 | 1,02347974 | ESTROGEN_RESPONSE_LATE |
| QSOX1 | 0,04116831 | 1,0233951 | EPITHELIAL_MESENCHYMAL_TRANSITION |
| MTHFD1 | 0,02027666 | 1,02324437 | XENOBIOTIC_METABOLISM |
| CDK2 | 0,03640861 | 1,02298128 | UV_RESPONSE_UP |
| FN1 | 0,0321162 | 1,02293514 | EPITHELIAL_MESENCHYMAL_TRANSITION |
| FN1 | 0,0321162 | 1,02293514 | COMPLEMENT |
| ENTPD5 | 0,00674278 | 1,02288685 | XENOBIOTIC_METABOLISM |
| PC | 0,02479368 | 1,02215149 | XENOBIOTIC_METABOLISM |
| JAG1 | 0,0321162 | 1,02210876 | ANGIOGENESIS |
| DHRS1 | 0,01767178 | 1,02207052 | XENOBIOTIC_METABOLISM |
| DHRS1 | 0,01767178 | 1,02207052 | XENOBIOTIC_METABOLISM |
| DLG4 | 0,02647704 | 1,02083796 | UV_RESPONSE_UP |
| PPARD | 0,03420622 | 1,02069065 | XENOBIOTIC_METABOLISM |
| GRB2 | 0,04926421 | 1,02043722 | COMPLEMENT |
| ABHD2 | 0,01331078 | 1,02000993 | ESTROGEN_RESPONSE_EARLY |
| ABHD2 | 0,01331078 | 1,02000993 | ESTROGEN_RESPONSE_LATE |
| MED24 | 0,01430322 | 1,01998531 | ESTROGEN_RESPONSE_EARLY |
| TMBIM6 | 0,02825584 | 1,01961496 | UV_RESPONSE_UP |
| TMBIM6 | 0,02825584 | 1,01961496 | XENOBIOTIC_METABOLISM |
| ADAM19 | 0,02479368 | 1,01943902 | IL2_STAT5_SIGNALING |
| NOP2 | 0,03013416 | 1,01766706 | IL2_STAT5_SIGNALING |
| CD81 | 0,02169734 | 1,01723413 | IL2_STAT5_SIGNALING |
| LATS2 | 0,03872779 | 1,01613132 | INTERFERON_GAMMA_RESPONSE |
| SLC37A1 | 0,01648049 | 1,01551892 | ESTROGEN_RESPONSE_EARLY |
| BGN | 0,03640861 | 1,01526119 | EPITHELIAL_MESENCHYMAL_TRANSITION |
| ACOX3 | 0,04926421 | 1,0151828 | XENOBIOTIC_METABOLISM |
| DHX58 | 0,04643181 | 1,01485051 | INTERFERON_GAMMA_RESPONSE |
| RNF31 | 0,02825584 | 1,01328346 | INTERFERON_GAMMA_RESPONSE |
| RNF31 | 0,02825584 | 1,01328346 | INTERFERON_GAMMA_RESPONSE |
| PVR | 0,03013416 | 1,01311388 | EPITHELIAL_MESENCHYMAL_TRANSITION |
| PVR | 0,03013416 | 1,01311388 | INFLAMMATORY_RESPONSE |
| EMP2 | 0,04116831 | 1,01265816 | ESTROGEN_RESPONSE_LATE |
| F5 | 0,02825584 | 0,9901073 | COMPLEMENT |
| STK25 | 0,01893601 | 0,98875436 | UV_RESPONSE_UP |
| CREG1 | 0,04926421 | 0,98523018 | UV_RESPONSE_UP |
| MCM7 | 0,02027666 | 0,98348978 | EPITHELIAL_MESENCHYMAL_TRANSITION |
| RAP1GAP | 0,04643181 | 0,98342384 | XENOBIOTIC_METABOLISM |
| CSRP1 | 0,04926421 | 0,98288901 | COMPLEMENT |
| TPBG | 0,02320174 | 0,98243608 | ESTROGEN_RESPONSE_EARLY |
| TPBG | 0,02320174 | 0,98243608 | ESTROGEN_RESPONSE_EARLY |
| TPBG | 0,02320174 | 0,98243608 | ESTROGEN_RESPONSE_LATE |
| TPBG | 0,02320174 | 0,98243608 | ESTROGEN_RESPONSE_LATE |
| TPBG | 0,02320174 | 0,98243608 | INFLAMMATORY_RESPONSE |
| TPBG | 0,02320174 | 0,98243608 | INFLAMMATORY_RESPONSE |
| SIGMAR1 | 0,00414023 | 0,98214854 | UV_RESPONSE_UP |
| PEMT | 0,02320174 | 0,98208887 | XENOBIOTIC_METABOLISM |
| SIRT6 | 0,02825584 | 0,98203485 | COMPLEMENT |
| LAD1 | 0,02825584 | 0,98132396 | ESTROGEN_RESPONSE_EARLY |
| XDH | 0,02169734 | 0,9809679 | XENOBIOTIC_METABOLISM |
| SIAH2 | 0,02647704 | 0,98085878 | ESTROGEN_RESPONSE_EARLY |
| SIAH2 | 0,02647704 | 0,98085878 | ESTROGEN_RESPONSE_LATE |
| LRPAP1 | 0,02647704 | 0,98069187 | ANGIOGENESIS |
| ACAA1 | 0,0321162 | 0,98056391 | UV_RESPONSE_UP |
| TNFRSF18 | 0,02647704 | 0,98049453 | IL2_STAT5_SIGNALING |
| ROS1 | 0,00729386 | 0,97948354 | INFLAMMATORY_RESPONSE |
| DDC | 0,00674278 | 0,9789267 | XENOBIOTIC_METABOLISM |
| FOXC2 | 0,01430322 | 0,97890048 | EPITHELIAL_MESENCHYMAL_TRANSITION |
| ATOH8 | 0,03640861 | 0,97871044 | XENOBIOTIC_METABOLISM |
| SQSTM1 | 0,00065408 | 0,97847827 | UV_RESPONSE_UP |
| SQSTM1 | 0,00065408 | 0,97847827 | UV_RESPONSE_UP |
| INHBB | 0,04926421 | 0,97847613 | ALLOGRAFT_REJECTION |
| INHBB | 0,04926421 | 0,97847613 | ESTROGEN_RESPONSE_EARLY |
| FAH | 0,03640861 | 0,97794025 | IL2_STAT5_SIGNALING |
| FAH | 0,03640861 | 0,97794025 | XENOBIOTIC_METABOLISM |
| MLPH | 0,04373476 | 0,97787503 | ESTROGEN_RESPONSE_EARLY |
| SLCO2A1 | 0,0321162 | 0,97757653 | ANGIOGENESIS |
| ABCC3 | 0,04926421 | 0,97748319 | XENOBIOTIC_METABOLISM |
| KCNK5 | 0,01237833 | 0,97712848 | ESTROGEN_RESPONSE_EARLY |
| KCNK5 | 0,01237833 | 0,97712848 | ESTROGEN_RESPONSE_LATE |
| WWC1 | 0,0029433 | 0,97695277 | ESTROGEN_RESPONSE_EARLY |
| KCNH2 | 0,04643181 | 0,97673999 | UV_RESPONSE_UP |
| ADH5 | 0,02479368 | 0,9761119 | XENOBIOTIC_METABOLISM |
| CDH1 | 0,02825584 | 0,97519868 | ESTROGEN_RESPONSE_LATE |
| NDRG2 | 0,02169734 | 0,97514667 | XENOBIOTIC_METABOLISM |
| DCXR | 0,03013416 | 0,97493034 | ESTROGEN_RESPONSE_LATE |
| DCXR | 0,03013416 | 0,97493034 | XENOBIOTIC_METABOLISM |
| MSX1 | 0,01237833 | 0,9747123 | EPITHELIAL_MESENCHYMAL_TRANSITION |
| MSX1 | 0,01237833 | 0,9747123 | ANGIOGENESIS |
| MSX1 | 0,01237833 | 0,9747123 | UV_RESPONSE_UP |
| MYCN | 0,04116831 | 0,97469647 | KRAS_SIGNALING_UP |
| KLK11 | 0,01767178 | 0,97461485 | ESTROGEN_RESPONSE_LATE |
| IL17RB | 0,04643181 | 0,97437289 | ESTROGEN_RESPONSE_EARLY |
| IL17RB | 0,04643181 | 0,97437289 | ESTROGEN_RESPONSE_LATE |
| PGRMC1 | 0,04643181 | 0,97430029 | XENOBIOTIC_METABOLISM |
| PRNP | 0,01331078 | 0,97414673 | IL2_STAT5_SIGNALING |
| CLTB | 0,01648049 | 0,9737442 | UV_RESPONSE_UP |
| FOXC1 | 0,00107314 | 0,97374365 | ESTROGEN_RESPONSE_EARLY |
| FOXC1 | 0,00107314 | 0,97374365 | ESTROGEN_RESPONSE_LATE |
| TSPAN1 | 0,04926421 | 0,97274637 | KRAS_SIGNALING_UP |
| HMGCS2 | 0,00851538 | 0,9725627 | ESTROGEN_RESPONSE_LATE |
| HPCAL4 | 0,01535873 | 0,9724157 | COMPLEMENT |
| AREG | 0,04926421 | 0,97228309 | EPITHELIAL_MESENCHYMAL_TRANSITION |
| AREG | 0,04926421 | 0,97228309 | ESTROGEN_RESPONSE_EARLY |
| AREG | 0,04926421 | 0,97228309 | ESTROGEN_RESPONSE_LATE |
| SEMA3B | 0,00188536 | 0,97173735 | ESTROGEN_RESPONSE_EARLY |
| SEMA3B | 0,00188536 | 0,97173735 | ESTROGEN_RESPONSE_LATE |
| SEMA3B | 0,00188536 | 0,97173735 | KRAS_SIGNALING_UP |
| ANKH | 0,00991168 | 0,97172446 | KRAS_SIGNALING_UP |
| BLVRB | 0,01331078 | 0,97157465 | ESTROGEN_RESPONSE_EARLY |
| BLVRB | 0,01331078 | 0,97157465 | ESTROGEN_RESPONSE_LATE |
| BLVRB | 0,01331078 | 0,97157465 | XENOBIOTIC_METABOLISM |
| PDCD2L | 0,00088285 | 0,97121144 | IL2_STAT5_SIGNALING |
| SLC22A5 | 0,02479368 | 0,97118574 | ESTROGEN_RESPONSE_EARLY |
| SLC22A5 | 0,02479368 | 0,97118574 | ESTROGEN_RESPONSE_LATE |
| COCH | 0,02320174 | 0,97066683 | IL2_STAT5_SIGNALING |
| SOX9 | 0,02320174 | 0,97031559 | KRAS_SIGNALING_UP |
| LTF | 0,04373476 | 0,97027346 | COMPLEMENT |
| LTF | 0,04373476 | 0,97027346 | ESTROGEN_RESPONSE_LATE |
| ACHE | 0,02825584 | 0,97023988 | ALLOGRAFT_REJECTION |
| ANGPTL3 | 0,03872779 | 0,97015622 | XENOBIOTIC_METABOLISM |
| NPTXR | 0,00414023 | 0,97011209 | UV_RESPONSE_UP |
| CACNA2D2 | 0,0014282 | 0,97001389 | ESTROGEN_RESPONSE_LATE |
| IL1RL2 | 0,02479368 | 0,96988167 | KRAS_SIGNALING_UP |
| LHX2 | 0,01430322 | 0,96944907 | UV_RESPONSE_UP |
| MYB | 0,00380637 | 0,96901828 | ESTROGEN_RESPONSE_EARLY |
| MYB | 0,00380637 | 0,96901828 | ESTROGEN_RESPONSE_LATE |
| S100A1 | 0,03013416 | 0,96878395 | IL2_STAT5_SIGNALING |
| VIP | 0,04373476 | 0,9683353 | INFLAMMATORY_RESPONSE |
| GADD45A | 0,03872779 | 0,96830017 | EPITHELIAL_MESENCHYMAL_TRANSITION |
| CD82 | 0,00488654 | 0,96822843 | INFLAMMATORY_RESPONSE |
| PTGIR | 0,00919046 | 0,96820362 | INFLAMMATORY_RESPONSE |
| TACR1 | 0,01767178 | 0,96788438 | INFLAMMATORY_RESPONSE |
| ALDH3A1 | 0,0017202 | 0,96761828 | XENOBIOTIC_METABOLISM |
| GYPC | 0,01767178 | 0,96761565 | KRAS_SIGNALING_UP |
| KRT19 | 0,00729386 | 0,96761295 | ESTROGEN_RESPONSE_EARLY |
| KRT19 | 0,00729386 | 0,96761295 | ESTROGEN_RESPONSE_LATE |
| SERPINC1 | 0,03640861 | 0,96733175 | COMPLEMENT |
| SERPINC1 | 0,03640861 | 0,96733175 | IL2_STAT5_SIGNALING |
| CCNE1 | 0,01430322 | 0,96710504 | IL2_STAT5_SIGNALING |
| CCNE1 | 0,01430322 | 0,96710504 | UV_RESPONSE_UP |
| FGL2 | 0,01430322 | 0,96682136 | IL2_STAT5_SIGNALING |
| FGL2 | 0,01430322 | 0,96682136 | INTERFERON_GAMMA_RESPONSE |
| MMP10 | 0,04116831 | 0,96617595 | KRAS_SIGNALING_UP |
| IGFBP1 | 0,00991168 | 0,96615314 | XENOBIOTIC_METABOLISM |
| CYP2J2 | 0,00246929 | 0,96590509 | XENOBIOTIC_METABOLISM |
| MEP1A | 0,02169734 | 0,96578968 | INFLAMMATORY_RESPONSE |
| PROK2 | 0,01535873 | 0,96497247 | INFLAMMATORY_RESPONSE |
| APOH | 0,02169734 | 0,96488287 | ANGIOGENESIS |
| NKX2-5 | 0,01430322 | 0,96475941 | UV_RESPONSE_UP |
| F11 | 0,00022542 | 0,9642728 | XENOBIOTIC_METABOLISM |
| ISOC1 | 0,00188536 | 0,96367028 | INTERFERON_GAMMA_RESPONSE |
| TMEM100 | 0,02027666 | 0,96315129 | KRAS_SIGNALING_UP |
| ESR1 | 0,00488654 | 0,9628728 | XENOBIOTIC_METABOLISM |
| CPE | 0,01767178 | 0,96276892 | ESTROGEN_RESPONSE_LATE |
| CPE | 0,01767178 | 0,96276892 | KRAS_SIGNALING_UP |
| NPFFR2 | 0,01767178 | 0,96269231 | INFLAMMATORY_RESPONSE |
| PEG3 | 0,04643181 | 0,96177911 | KRAS_SIGNALING_UP |
| CXCL2 | 0,03420622 | 0,96166775 | UV_RESPONSE_UP |
| HES6 | 0,02647704 | 0,96155953 | XENOBIOTIC_METABOLISM |
| OPRK1 | 0,00156811 | 0,96138065 | INFLAMMATORY_RESPONSE |
| WNT7A | 0,00622855 | 0,96091065 | KRAS_SIGNALING_UP |
| MAPK8IP2 | 0,00269704 | 0,9609016 | UV_RESPONSE_UP |
| SPR | 0,00851538 | 0,9607632 | UV_RESPONSE_UP |
| MRPL23 | 0,04116831 | 0,96063919 | UV_RESPONSE_UP |
| MRPL23 | 0,04116831 | 0,96063919 | UV_RESPONSE_UP |
| C4BPB | 0,02169734 | 0,96023204 | COMPLEMENT |
| C4BPB | 0,02169734 | 0,96023204 | UV_RESPONSE_UP |
| SMOX | 0,0106816 | 0,95939446 | XENOBIOTIC_METABOLISM |
| CRP | 0,01767178 | 0,95932442 | XENOBIOTIC_METABOLISM |
| SERPINB2 | 0,00206457 | 0,95922737 | COMPLEMENT |
| ARG1 | 0,00788397 | 0,95811418 | KRAS_SIGNALING_UP |
| ARG1 | 0,00788397 | 0,95811418 | XENOBIOTIC_METABOLISM |
| TMEM97 | 0,00380637 | 0,95797581 | XENOBIOTIC_METABOLISM |
| CYP2C18 | 0,00107314 | 0,95734876 | XENOBIOTIC_METABOLISM |
| HOPX | 0,00072355 | 0,95728965 | IL2_STAT5_SIGNALING |
| SOCS1 | 0,04926421 | 0,95713522 | ALLOGRAFT_REJECTION |
| SOCS1 | 0,04926421 | 0,95713522 | IL2_STAT5_SIGNALING |
| SOCS1 | 0,04926421 | 0,95713522 | INTERFERON_GAMMA_RESPONSE |
| GALM | 0,0321162 | 0,95713273 | IL2_STAT5_SIGNALING |
| MAP7 | 0,02320174 | 0,95700132 | KRAS_SIGNALING_UP |
| CKB | 0,02647704 | 0,95671198 | ESTROGEN_RESPONSE_LATE |
| SLC19A2 | 0,04643181 | 0,95571068 | ESTROGEN_RESPONSE_EARLY |
| GFRA1 | 0,04926421 | 0,95464626 | ESTROGEN_RESPONSE_EARLY |
| SFN | 0,00188536 | 0,95459351 | ESTROGEN_RESPONSE_EARLY |
| SFN | 0,00188536 | 0,95459351 | ESTROGEN_RESPONSE_LATE |
| IL12B | 0,00246929 | 0,95354325 | ALLOGRAFT_REJECTION |
| IL12B | 0,00246929 | 0,95354325 | INFLAMMATORY_RESPONSE |
| SHOX2 | 0,00065408 | 0,95333787 | UV_RESPONSE_UP |
| SLC27A2 | 0,01535873 | 0,95327909 | ESTROGEN_RESPONSE_EARLY |
| SLC27A2 | 0,01535873 | 0,95327909 | ESTROGEN_RESPONSE_LATE |
| SPARCL1 | 0,00156811 | 0,95325072 | KRAS_SIGNALING_UP |
| MSMB | 0,04116831 | 0,95306494 | ESTROGEN_RESPONSE_EARLY |
| GAD1 | 0,02825584 | 0,952356 | XENOBIOTIC_METABOLISM |
| PCSK1N | 0,01150289 | 0,94983598 | KRAS_SIGNALING_UP |
| KCNJ8 | 0,00530238 | 0,94914836 | ANGIOGENESIS |
| IL13 | 0,01237833 | 0,94914556 | ALLOGRAFT_REJECTION |
| IL13 | 0,01237833 | 0,94914556 | IL2_STAT5_SIGNALING |
| PTHLH | 0,00206457 | 0,94803638 | EPITHELIAL_MESENCHYMAL_TRANSITION |
| PCOLCE2 | 0,02479368 | 0,94774204 | EPITHELIAL_MESENCHYMAL_TRANSITION |
| KCNK15 | 0,00025195 | 0,94763379 | ESTROGEN_RESPONSE_EARLY |
| CLU | 0,00269704 | 0,94754262 | COMPLEMENT |
| TSKU | 0,00991168 | 0,9470331 | ESTROGEN_RESPONSE_EARLY |
| VWA5A | 0,00349658 | 0,94664836 | KRAS_SIGNALING_UP |
| FBN2 | 0,01331078 | 0,9451106 | EPITHELIAL_MESENCHYMAL_TRANSITION |
| NDP | 0,03013416 | 0,94421833 | INFLAMMATORY_RESPONSE |
| MCCC2 | 0,01535873 | 0,9439742 | XENOBIOTIC_METABOLISM |
| MCCC2 | 0,01535873 | 0,9439742 | XENOBIOTIC_METABOLISM |
| MCCC2 | 0,01535873 | 0,9439742 | XENOBIOTIC_METABOLISM |
| KIF5C | 0,00349658 | 0,94348221 | KRAS_SIGNALING_UP |
| KIF5C | 0,00349658 | 0,94348221 | KRAS_SIGNALING_UP |
| PCLO | 0,00622855 | 0,94332061 | COMPLEMENT |
| ASCL1 | 0,03640861 | 0,94276545 | ESTROGEN_RESPONSE_LATE |
| WFS1 | 0,00991168 | 0,94269137 | ESTROGEN_RESPONSE_EARLY |
| WFS1 | 0,00991168 | 0,94269137 | ESTROGEN_RESPONSE_LATE |
| EIF5A | 0,00020146 | 0,94055019 | ALLOGRAFT_REJECTION |
| EIF5A | 0,00020146 | 0,94055019 | ALLOGRAFT_REJECTION |
| HSPA4L | 0,02825584 | 0,93946342 | ESTROGEN_RESPONSE_LATE |
| NPTX2 | 0,00246929 | 0,9369413 | UV_RESPONSE_UP |
| PHEX | 0,00088285 | 0,93684922 | COMPLEMENT |
| AKR1C3 | 0,00488654 | 0,9359843 | XENOBIOTIC_METABOLISM |
| PRKAR2B | 0,02825584 | 0,93534481 | ESTROGEN_RESPONSE_LATE |
| PRKAR2B | 0,02825584 | 0,93534481 | ESTROGEN_RESPONSE_LATE |
| COMP | 0,01430322 | 0,93438877 | EPITHELIAL_MESENCHYMAL_TRANSITION |
| AKR1C2 | 0,00729386 | 0,93391189 | XENOBIOTIC_METABOLISM |
| MPPED2 | 0,03872779 | 0,93069973 | ESTROGEN_RESPONSE_EARLY |
| OLFML3 | 0,00107314 | 0,93038592 | ESTROGEN_RESPONSE_EARLY |
| AOX1 | 0,00414023 | 0,92948611 | XENOBIOTIC_METABOLISM |
| GADD45G | 0,000389 | 0,92879255 | KRAS_SIGNALING_UP |
| LAMA2 | 0,01535873 | 0,92801915 | EPITHELIAL_MESENCHYMAL_TRANSITION |
| PRKG2 | 5,09E-07 | 0,92358177 | KRAS_SIGNALING_UP |
| CDO1 | 0,00097381 | 0,92032432 | UV_RESPONSE_UP |
| CDO1 | 0,00097381 | 0,92032432 | XENOBIOTIC_METABOLISM |
| NAP1L2 | 0,01535873 | 0,9196752 | KRAS_SIGNALING_UP |
| SLC39A8 | 0,00072355 | 0,91795003 | IL2_STAT5_SIGNALING |
| ACTN2 | 0,00107314 | 0,91354027 | COMPLEMENT |
| ENPP1 | 0,00414023 | 0,90453171 | IL2_STAT5_SIGNALING |
| BTC | 0,00017984 | 0,89702603 | KRAS_SIGNALING_UP |
| PTPRD | 0,02027666 | 0,89018282 | UV_RESPONSE_UP |
| PTPRD | 0,02027666 | 0,89018282 | UV_RESPONSE_UP |
| RBP4 | 0,00246929 | 0,88974911 | KRAS_SIGNALING_UP |
| RBP4 | 0,00246929 | 0,88974911 | XENOBIOTIC_METABOLISM |
| CARTPT | 0,00107314 | 0,61842735 | ALLOGRAFT_REJECTION |

Non-dilated (nonDIL) aorta <40mm; Dilated (DIL) aorta >45mm.

**Supplementary Table 4.** Differentially expressed putative transcription factors (aortic intima-media) within HiCap distals, sorted by fold-change.

| **TF Gene** | **P (DIL/nonDIL)** | **Fc (DIL/nonDIL)** |
| --- | --- | --- |
| ZNF675 | 0.0153587278389084 | 1.1447230524131 |
| FOS | 0.148732958785156 | 1.06779446736275 |
| ZNF28 | 0.0620438940999948 | 1.0621757067364 |
| EGR1 | 0.0620438940999948 | 1.06055814015218 |
| ETS1 | 0.00246929404189283 | 1.05520891051488 |
| TCF7L2 | 0.00622855242723414 | 1.04791125218166 |
| STAT1 | 0.117007251070308 | 1.03508879605053 |
| ZNF571 | 0.135349219568088 | 1.03249600688746 |
| SP2 | 0.0106816000232584 | 1.03128343139877 |
| STAT3 | 0.0164804859148546 | 1.02487089306509 |
| MAX | 0.024793675747373 | 1.02296105849508 |
| FOXJ2 | 0.260494930399728 | 1.02098415001323 |
| RELA | 0.129008489440418 | 1.02043225792588 |
| CTCF | 0.221323577817937 | 1.01949026867172 |
| RXRA | 0.100636656435278 | 1.0159419957753 |
| USF2 | 0.476508566366397 | 1.0118594662156 |
| RREB1 | 0.230705055077948 | 1.0111059586714 |
| PLAGL2 | 0.726746866406361 | 1.01073962443197 |
| MEIS1 | 0.638267659021733 | 1.00891767470659 |
| FOXJ3 | 0.837731720348191 | 1.00873549943177 |
| PRRX1 | 0.587412578365279 | 1.00762907423966 |
| RHOXF1 | 0.352210860728928 | 1.00716017604415 |
| ZEB1 | 0.971174518067612 | 1.00667716339391 |
| TAF1 | 1 | 1.00589712711169 |
| FOXL1 | 0.292785636119094 | 1.0028345682899 |
| POU2F2 | 0.856606043887873 | 1.0021205807881 |
| HNF4A | 0.875560975775117 | 1.00078104638587 |
| NR2C2 | 0.894586057015542 | 1.00063623323086 |
| GATA5 | 0.818948370693141 | 1.00040019724383 |
| JDP2 | 0.932804155694214 | 1.00021243811933 |
| PRRX2 | 0.875560975775117 | 0.994159297101699 |
| MIXL1 | 0.587412578365279 | 0.993641143594618 |
| ESX1 | 0.781695179060942 | 0.993275307034537 |
| ZIM3 | 0.638267659021733 | 0.99314127871882 |
| NR2F1 | 0.600606610179292 | 0.991831220927442 |
| ZXDB | 0.221323577817937 | 0.990815484683309 |
| SALL2 | 0.39121199259741 | 0.990281899539025 |
| FOXP3 | 0.31571426883821 | 0.989703262435903 |
| VSX2 | 0.377935695319099 | 0.989451573650116 |
| MEIS3 | 0.240359504828503 | 0.987544258386532 |
| GATA2 | 0.148732958785156 | 0.987154816757437 |
| MEIS2 | 0.327599720274697 | 0.985661065052074 |
| FOXO1 | 0.0522367401981544 | 0.984968514528177 |
| ZBTB44 | 0.304109540894158 | 0.984403654261239 |
| FOXO4 | 0.0264770398767523 | 0.983988903785496 |
| LHX9 | 0.250288902621708 | 0.983801778081812 |
| LMX1A | 0.178424992488391 | 0.982177719088309 |
| VAX2 | 0.041168311458487 | 0.982038148846533 |
| DLX5 | 0.292785636119094 | 0.981656287735247 |
| GATA4 | 0.129008489440418 | 0.981480146164868 |
| VAX1 | 0.270979024116657 | 0.981340834787174 |
| PITX1 | 0.100636656435278 | 0.980339159573221 |
| NFE2 | 0.404761498986948 | 0.979717071566114 |
| FOSL1 | 0.364934735932384 | 0.979588216215175 |
| PDX1 | 0.0656259243997432 | 0.979059196161298 |
| ISL2 | 0.0732893900660821 | 0.978568991648 |
| KLF17 | 0.0620438940999948 | 0.977729036632133 |
| DLX2 | 0.0553542579277356 | 0.977116298949453 |
| GSC | 0.041168311458487 | 0.976610146018688 |
| BARHL2 | 0.0816522361787568 | 0.97658626291171 |
| HOXB3 | 0.240359504828503 | 0.976562630954397 |
| FOXI1 | 0.0907546718489362 | 0.976254798612721 |
| MSX1 | 0.0123783334200531 | 0.974712296009362 |
| FOXD3 | 0.0342062224096652 | 0.974397795616435 |
| ZXDA | 0.0387277924404637 | 0.974109154689703 |
| OTX2 | 0.117007251070308 | 0.973990679825999 |
| FIGLA | 0.0189360067903505 | 0.973487566779041 |
| FOXA1 | 0.041168311458487 | 0.972293217259641 |
| LBX2 | 0.148732958785156 | 0.971913294095852 |
| HOXB2 | 0.0816522361787568 | 0.970984915716607 |
| RAX2 | 0.0732893900660821 | 0.970515857185599 |
| EN1 | 0.0342062224096652 | 0.970302165560565 |
| GBX2 | 0.0106816000232584 | 0.969078204299461 |
| MNX1 | 0.00851537802609044 | 0.968136079522308 |
| TBPL2 | 0.0321161967134646 | 0.967884862565888 |
| PITX3 | 0.0202766574952346 | 0.965530239617616 |
| FOXD2 | 0.0164804859148546 | 0.965010876052144 |
| HOXB5 | 0.0522367401981544 | 0.964003194893233 |
| FOXO3 | 0.00294330467390974 | 0.953910891272732 |
| SHOX2 | 0.000654078736299039 | 0.953337874870574 |
| CPEB1 | 0.0014282014964061 | 0.950696959198565 |
| GSC2 | 0.000723550647869406 | 0.948767992564255 |

Non-dilated (nonDIL) aorta <40mm; Dilated (DIL) aorta >45mm.

**Supplementary Figure S1. Enrichment analysis of differentially expressed genes**. Number of differentially expressed genes between non-dilated and dilated aortas in BAV and TAV patients, respectively, and their enrichment in Hallmarks. Hallmarks retrieved from ‘Molecular Signatures Database’ (MSigDB, v7.5) using the msigdbr package. Significance is color-coded from red to green, for lower and higher significant p-values, respectively.

**
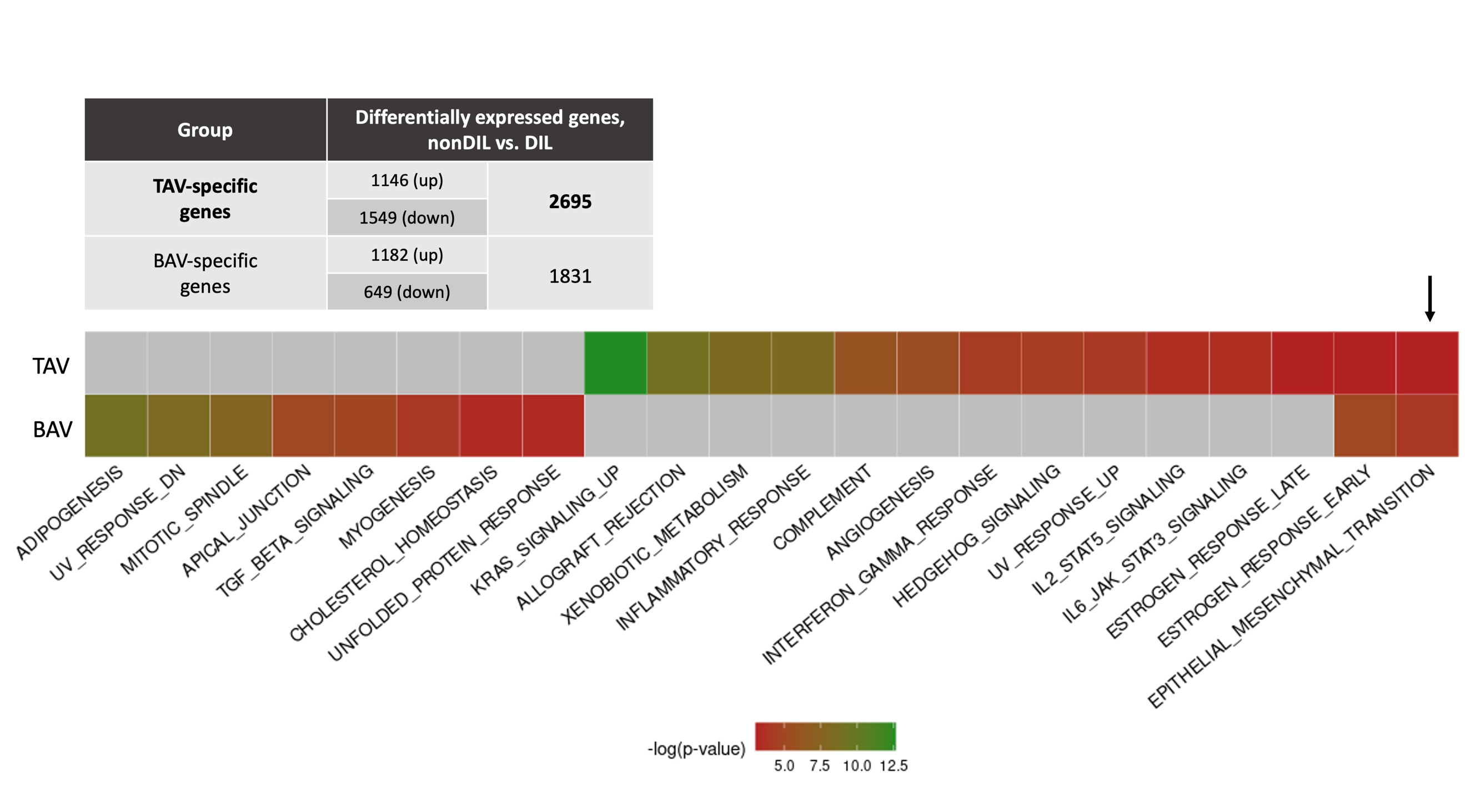
**

**Supplementary Figure S2. Putative transcription factors (TF) within each HiCap-identified distal**. TRANSFAC^®^ was used to identify putative TFs. Genes within each distal fragment are denoted with corresponding color to the distal they were identified in.


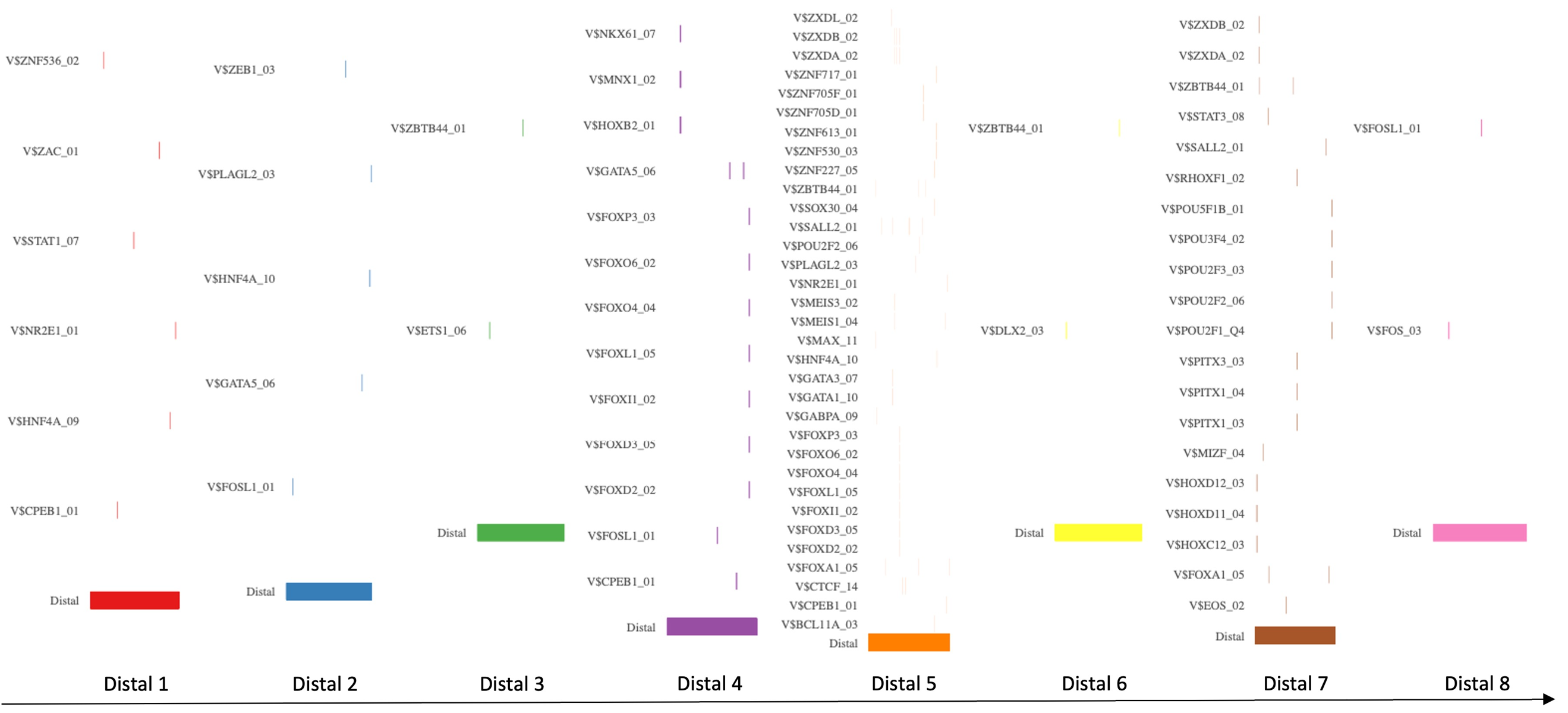

Supplement: Supplementary file 1 — Supplementary file1 (DOCX 1759 KB) [file 109_2023_2370_MOESM1_ESM.docx]
